# Raman Optical Activity as a Tool to Monitor Polynucleotide Conformation in Solutions

**DOI:** 10.1002/chem.71445

**Published:** 2026-07-29

**Authors:** Mohammed Siddhique Para Kkadan, Josef Kapitán, Jiří Kessler, Ondřej Zapletal, Valery Andrushchenko, Petr Bouř

**Affiliations:** ^1^ Institute of Organic Chemistry and Biochemistry Academy of Sciences Prague Czech Republic; ^2^ Institute of Physics, Faculty of Mathematics and Physics Charles University Prague Czech Republic; ^3^ Department of Optics Faculty of Sciences Palacký University Olomouc Olomouc Czech Republic

**Keywords:** density functional calculations, molecular dynamics, nucleotides, Raman optical activity, temperature dependence

## Abstract

The reactivity and function of biopolymers depend on their structure and flexibility. There is a limited number of methods that can be used for their studies in solutions. Among them, Raman optical activity (ROA) provides excellent sensitivity to conformational changes. So far, ROA studies of nucleic acid systems are relatively rare because of the complexity of these molecules and difficulties in interpretation of the spectra. To explore the link between spectral shapes and the structure, and to advance the experimental and computational methodologies, we measured Raman and ROA spectra of four oligonucleotides (polyA, polyC, polyG, and polyU) in a wide wavenumber range. Molecular dynamics (MD) and density functional theory (DFT) were used for the spectra simulations. Temperature‐dependent vibrational spectral changes are consistent with melting curves obtained from electronic circular dichroism (ECD). The results show that the spectra well‐reflect molecular geometry, including changes caused by temperature variation. Comparison of theoretical and experimental Raman and ROA intensities appears as a convenient way to validate and potentially develop MD force fields; the RNA.Shaw force field provided results superior to the RNA.OL3 one. The combined spectroscopic and computational methodology thus can be used as a powerful means to study solution properties of nucleic acids.

## Graphical abstract

Raman optical activity (ROA) studies of nucleic acids have been hindered by limited computational tools to link the spectra to the structure. The study shows that accurate density functional simulations are possible for model nucleotides, bringing more information about molecular geometry and dynamics in solutions.

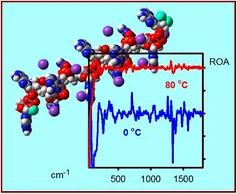

## 1 Introduction

Ribonucleotides are excellent models for DNA and RNA behavior. They are also omnipresent in living organisms. For example, polyadenylic acid (polyA) tails occur in eukaryotic messenger RNAs (mRNAs), containing up to 200 adenosine residues [1, 2]. They affect mRNA maturation, transport, stability, and translational efficiency [3], making them suitable targets for therapeutic interventions [4]. PolyA also occurs in some mRNA vaccines, such as those against the COVID‐19 flue, where it improves the stability and protein expression [5, 6].

Polycytidylic acid (polyC) is a part of viral RNA [7], its C‐rich regions sometimes fold into *i*‐motifs [8, 9, 10]. Similarly as polyC, polyguanylic acid (polyG) also forms specific secondary structures known as G‐quadruplexes [11, 12, 13, 14]. They are over‐represented in cancerous tissues [15], and catalyze RNA formation in the prebiotic stage [16, 17]. Both the *i*‐motifs and G‐quadruplexes attracted attention in nanotechnology and chemical biology [9]. Polyribonucleotide tracts are recognized by a range of RNA‐binding proteins, such as the nuclear polyA‐binding proteins regulating the transcription or cytoplasmic polyA‐binding proteins involved in interactions with a ribosome [3]. The α‐complex proteins bind to polyC, and the fragile X mental retardation proteins selectively bind to polyG and polyU chains [18, 19].

Various methods have been used to study polynucleotides in solutions, including NMR [20] and cryoEM [21]. Vibrational spectroscopy gives a lower spatial resolution but is very flexible, relatively cheap, fast, and nondestructive. Infrared (IR) and Raman spectroscopy provided precious information on nucleotide conformations, base‐stacking interactions, sugar puckering, and backbone geometry [22, 23, 24, 25]. Vibrational optical activity comprising vibrational circular dichroism (VCD) and Raman optical activity (ROA) offers even greater sensitivity to structural details than unpolarized IR and Raman. VCD provides information mostly about the base stacking and pairing [26, 27, 28, 29, 30, 31, 32]. ROA monitors wider range of vibrational frequencies and adds sensitivity to the sugar‐phosphate backbone [33, 34, 35, 36, 37].

Compared to peptides and proteins [38], ROA studies of nucleic acid components are more challenging. Nucleic acids are less stable, may decompose in the excitation laser beam, or exhibit solubility problems [39]. Nevertheless, basic spectral features of single‐stranded nucleotides have been determined [33]. Later, even temperature‐induced ROA spectral changes for nucleotides, RNA and DNA were detected [36, 40]. An interesting chapter in ROA spectroscopy development was the measurement of whole viruses [41, 42, 43].

Many problems are also encountered in the interpretation of polynucleotide ROA spectra. While VCD of nucleic acids could be reasonably described in terms of a simple coupled oscillator model [44, 45], ROA signals are more complex, typically requiring quantum‐chemical computations for the interpretation. Although simulations of vibrational optical activity are nowadays possible within density functional theory (DFT) [46, 47, 48], they may be prohibitively demanding when applied to larger systems. In the present work, we therefore use the Cartesian‐coordinate tensor (CCT) transfer techniques [49, 50, 51] that allow one to extend the calculations to macromolecular systems up to about 10000 atoms, both for VCD [52] and ROA [53]. Further extension is possible through exploration of system periodicity [54].

The flexibility of nucleic acids makes their structural studies difficult as well. While the secondary structure of proteins is mostly determined by two torsion angles (*φ* and *ψ*), seven analogous angles (*α, β, γ, δ, ε, ζ*, and *χ*) form the polynucleotide backbone. In addition, the polymer unit is larger: an amino acid typically contains about 20 atoms, against about 35 of them in a mononucleotide. This makes a big difference in DFT frequency computations, where the time may scale with the fifth power of the number of atoms.

Finally, nucleotide building blocks—sugars, phosphates, and the bases—have rather different chemical properties, which again complicates their modeling. For example, while the calculated vibrational frequencies are usually higher than the experimental ones, the theoretical P═O stretching frequency may be significantly lower [35]. An incorrect vibrational frequency ordering in particular affects ROA, where it can lead to wrong band assignments.

To address these experimental and theoretical challenges, we acquired original Raman and ROA spectra of selected polyribonucleotides on a sensitive spectrometer [55]. It operates approximately within the 50–4500 cm^−1^ range, much larger than in previous studies typically covering about 700–1800 cm^−1^ [36, 40]. Several molecular dynamics (MD) force fields were used to reproduce the polynucleotide behavior, and spectral intensities were simulated at the DFT level combined with CCT.

Homopolyribonucleotides polyA, polyC, polyG, and polyU were selected because of their biological relevance and relative simplicity for modeling. As shown below, Raman and ROA spectroscopy indeed appeared to well reflect the solution structure, including temperature‐induced changes, and the simulations allowed us to establish a link between the spectral features and the structure. The results are thus encouraging for usage of vibrational spectroscopy techniques for more advanced studies of nucleic acids in their natural aqueous environment.

## 2 Results and Discussion

### 2.1 Molecular Dynamics

To understand molecular behavior and the spectroscopic properties, MD was performed within the Amber software package [56]. Initial geometries of all polynucleotides (Figure 1) were obtained from the Protein Data Bank, standard A‐DNA torsion angles were used for initial geometry, except for polyG adopting a G‐quadruplex [57, 58]. Further computational details can be found in Supporting Information.

**FIGURE 1:**
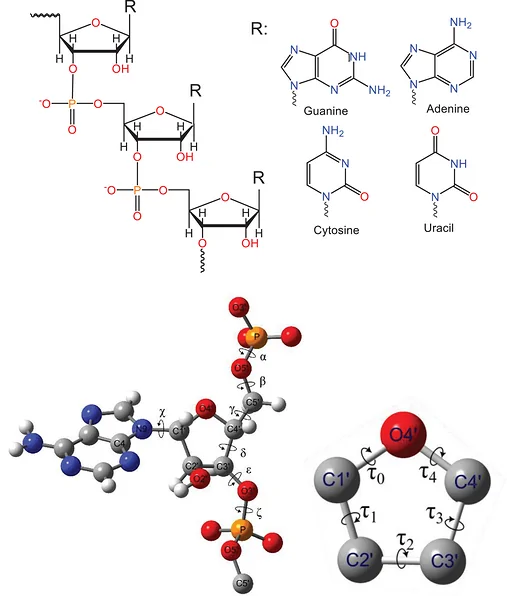
Structure of the polyribonucleotides, and definitions of the main chain and sugar ring torsion angles. The sugar puckering (*P*) is defined from tan(*P*) = (τ_4_ + τ_1_—‐τ_3_ ‐ τ_0_)/{2 × τ_2_ × [sin(36)+sin(72)]} [59].

Most geometry parameters as obtained from MD using the RNA.Shaw force field are summarized in Table 1, probability distributions of the puckering (*P*) and glycosidic angle (*χ*) are plotted in Figure 2, top. The average values are fairly close to the parameters of standard A‐DNA conformation, even for the polyG quadruplex. This is in line with known behavior of the polynucleotides. For example, at physiological pH and temperature, polyA was found to adopt A‐form characterized by a C3'‐endo sugar pucker and *anti*‐glycosidic torsion angle [27]. For polyA, this conformation is stabilized by adenine‐adenine π–π electron stacking and hydrogen bonds between adjacent adenine NH_2_ groups [18]. Compared to polyA and polyC, in polyU more residues adopt the C2'‐endo form, and the backbone parameters vary more as well.

**TABLE 1:** Most probable torsion angle and sugar pucker values (in degrees), relative conformer ratios (in %, in [] brackets) obtained from MD, and standard DNA/RNA values.

	Molecular dynamics				Standard [60, 61, 62]	
	PolyA	PolyU	PolyC	PolyG	B‐conf.	A‐conf.
*α*	−75	−77, 66 [50, 23]	−74.5	−72, 58 [57, 41]	−30	−65
*β*	175	176	174	171	136	173
*γ*	60	58	58	61	31	54
*δ*	79	77, 140 [72, 27]	79	77, 146 [63, 37]	143	80
*ε*	−154	−169, −88 [76, 23]	−145	−162, −76 [67, 33]	−141	−150
*ζ*	−62	−83, 80 [66, 27]	−57	−71, 80 [57, 37]	−161	−73
*χ*	−167	−147	−164	−163, −137	−98	−161
*P*	11	22, 153 [71, 28]	15	10, 166 [63, 37]	150	5

**FIGURE 2:**
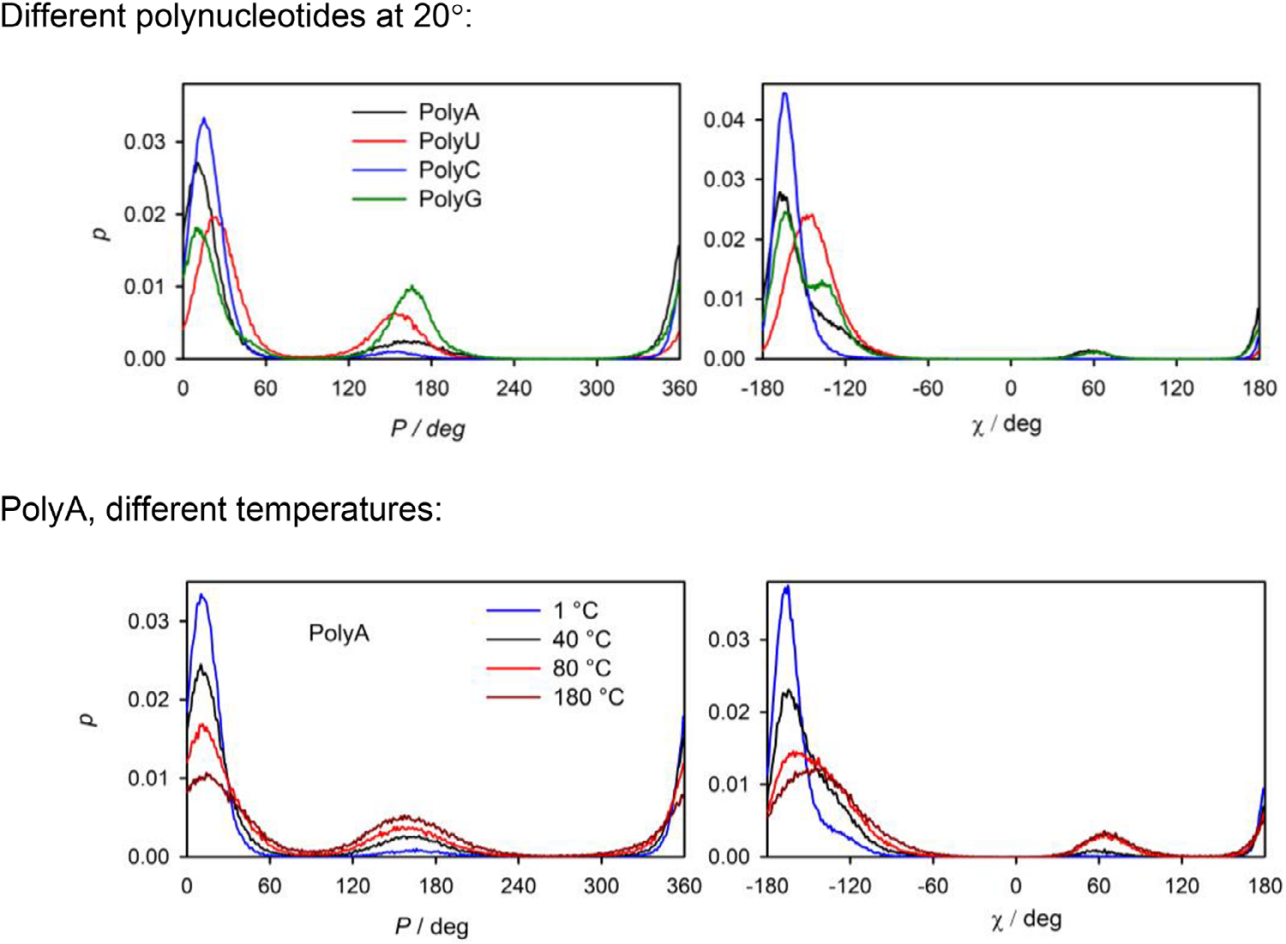
Examples of MD probabilities (*p*) of sugar pucker (*P*) and glycosidic torsion angle (*χ*) as obtained from MD.

At higher temperatures the distributions for polyA are broader, and populations of different conformations (C3'‐endo and C2'‐endo, *syn*, and *anti* of χ) are more even (Figure 2, bottom). For polyU and polyC, the temperature had a very similar effect (Figure S2).

For the G‐quadruplex the distributions did not change much with the temperature. The polyG structure was also more rigid, the coordinate root mean square deviation, RMSD, was 0.4 Å, less than for polyA, polyU and polyC with RMSDs of 1.1–1.7 Å. However, during MD, the metal counterions intercalated between the G residues. This is consistent with previous experiments [63] and simulations [32, 64, 65], although it should be noted that the 2MB2 model is only one of many possible G‐quadruplex geometries, and we do not know which one corresponds best to our sample.

### 2.2 Spectra Measurement and Simulations

Raman and ROA spectra were recorded with a spectrometer located at the Palacký University in Olomouc [55], operating within 50–4500 cm^−1^, in the scattered circular polarization (SCP) modulation, backscattering geometry, and using 532 nm laser excitation (cf. Supporting Information). As an alternative method to monitor temperature‐dependent conformational changes, UV absorption and electronic circular dichroism (ECD) spectra were obtained at several temperatures. From selected points of absorption, ECD, Raman, and ROA spectra recorded for different temperatures “melting temperatures” *T_m_* were obtained by a fit, using a Boltzmann function [66],

$$S\left(T\right)=S_{\max}+\frac{S_{\min}-S_{\max}}{1+e^{(T-T_{m})/d}}$$

where *S*_min_ and *S*_max_ are limit values for low and high temperatures, respectively, and *d* is a parameter.

The vibrational spectra were simulated for 20 MD snapshot geometries including the first hydration sphere, and averaged. Using an automatic fragmentation procedure, similar to that used for peptides [53], the snapshot geometries were divided into overlapping trinucleotide units including the surrounding solvent molecules (Figure S1). Quantum chemical calculations on the fragments were carried out using the Gaussian 16 software package [67], the geometries were partially optimized in the normal mode coordinates [68, 69] and the Cartesian coordinate tensor transfer (CCT) [49, 50], was applied to generate the spectra for the polynucleotides. Since it is not possible to measure absolute Raman intensities, the simulated Raman and ROA intensities were multiplied by the same factor, making calculated and experimental Raman areas within 450–1800 cm^−1^ the same. This has been done for each sample measured, since it is difficult to accurately normalize experiments performed at different conditions. Note, however, that the factor is the same for Raman and ROA spectra, so that the simulated ROA/Raman ratio is not affected. For a fast comparison of the simulated and experimental spectra, similarities were calculated as $s=\int S_{sim}S_{\exp}d\nu /\sqrt{\int S_{sim}^{2}d\nu \int S_{\exp}^{2}d\nu }$, where the calculated frequencies were multiplied by a factor giving the highest *s*. The integrations ran within 450–1800 cm^−1^.

The spectra enable one to verify the theoretical models. Compared to the RNA.Shaw force field, the RNA.OL3 one leads to notably different geometry (e.g., the *P* and *χ* distributions for polyA, Figure S3) and, in final effect, to incorrect Raman and ROA spectra. This is shown in Figure 3 for polyA. One can see that while both the calculated Raman spectra reasonably reproduce the experimental one, RNA.Shaw provides occasional improvements, such as the better 1338/1304 band ratio. These differences are not captured in the similarities with respect to the experiments, which are nearly the same (0.90 vs. 0.89).

**FIGURE 3:**
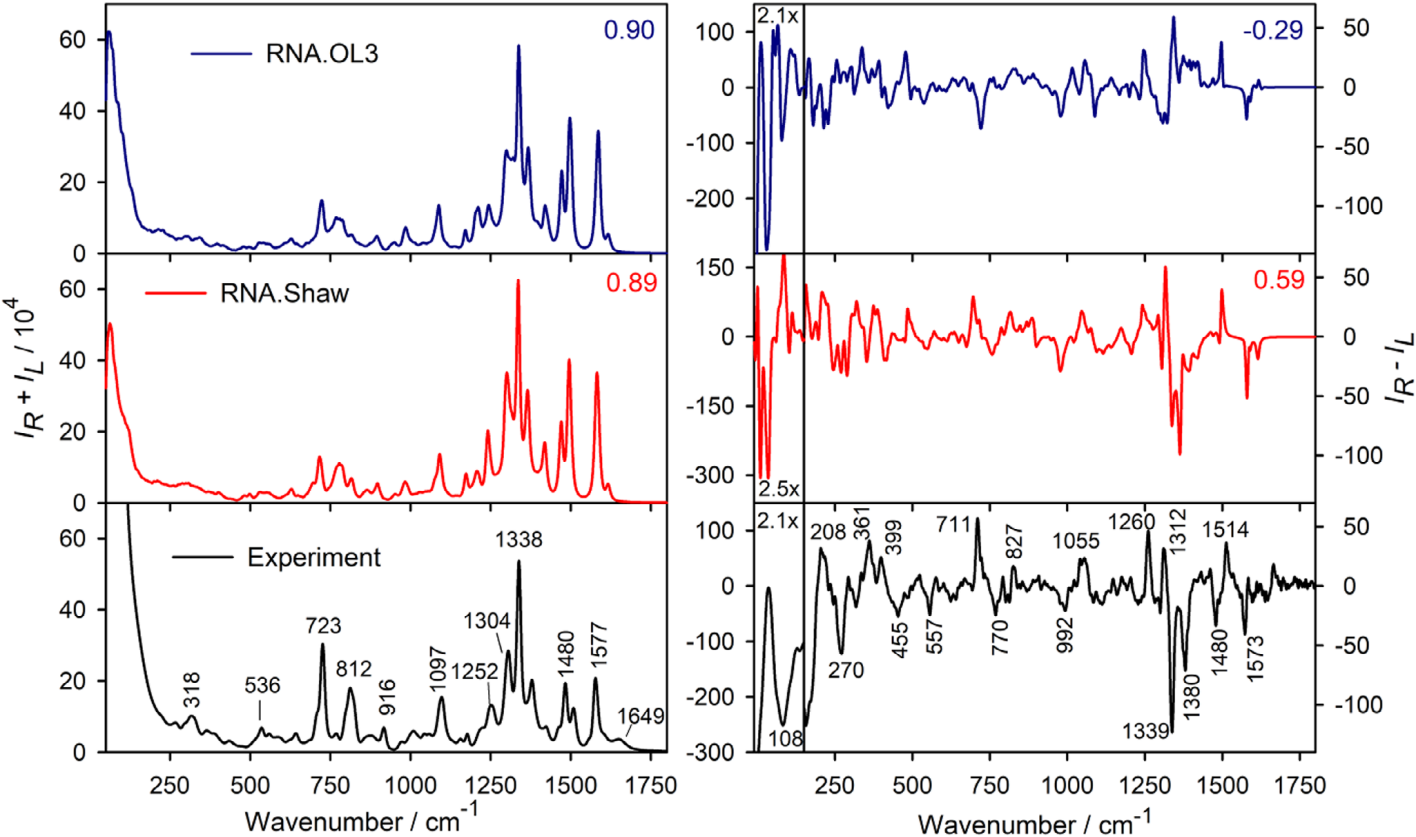
PolyA, Raman and ROA spectra calculated with the RNA.OL3 and RNA.Shaw force fields, and the experiment at 20°C. The spectra were computed for A_11_ with the CPCM solvation, ten MD snapshots were averaged. Similarities of the calculated spectra to the experiment are given in the theoretical panels, ROA spectra are plotted with separate intensity scales below and above 200 cm^−1^, indicated on the left and right axes.

The differences in ROA spectra are much more extensive than in Raman. The RNA.OL3 force field provides ROA spectrum very different from the experimental one, with a negative similarity index of ‐0.29. Most blatant discrepancies include the 1250–1500 cm^−1^ region (typically CH and OH bending modes, cf. the mode assignment in Table S1) and sugar‐base deformations around 700 cm^−1^, where the simulation gives mostly bands of opposite signs compared to the experiment. The RNA.Shaw results are much more realistic, giving a positive similarity index of 0.59. Below 200 cm^−1^, the simulations reproduce the large experimental ROA signal, but band‐to‐band assignment is not possible. For example, around 108 cm^−1^ a negative experimental signal prevails, while predominantly positive one is predicted by the calculation. In the theoretical spectrum strong negative bands appear at much lower (∼70 cm^−1^) frequencies. The inconsistencies in this region can be explained by a large participation of the solvent [70, 71], the necessity of nonoptimized vibrational coordinates to preserve the MD geometry in the constrained optimization [68, 69], anharmonic potential energy surface [72], and the presence of hot vibrational bands. None of these factors can be fully included in the present simulation protocol.

These results document the sensitivity of ROA spectroscopy to subtle conformational variations, not seen by unpolarized Raman spectroscopy. ROA spectroscopy clearly provides means to calibrate and validate new MD force fields. In this particular case, the RNA.Shaw force field preserved conformations closer to the canonical A‐RNA form, which appeared more realistic than the looser structure supported by RNA.OL3.

### 2.3 Solvent Effects on the Spectral Intensities

Several solvent effects could be examined in details using a computational model consisting of an adenosine dimer. The molecule was kept in the A‐conformation, to separate solvent influence from conformational dependencies. Raman and ROA spectra calculated in vacuum, using the dielectric CPCM environment, and a combined explicit+CPCM model are compared in Figure 4. For example, the symmetric P─O (single bond) stretching band is not much affected by the solvent. In Raman spectra, we see that this band is calculated in vacuum at 751 cm^−1^, it moves up to 755 cm^−1^ in the CPCM environment, and further up to 764 cm^−1^ for the explicit model. Since the frequency does not depend much on the nucleotide length, the model can be compared to polyA experiment, where this vibration is found at 810 cm^−1^ (Figure 3). To understand this relatively large difference, we calculated the frequency in the dimethylphosphate ion at several levels of approximation (Table S2). The differences suggest that a large part of the discrepancy comes from the DFT error; for example, the MP2/6‐311++G**/CPCM value is by 25 cm^−1^ higher than that from B3LYP/6‐311++G**/CPCM. The asymmetric P─O stretching is of lower intensity, quite close in frequency, and in A_2_ and polyA likely overlapped with the symmetric signal.

**FIGURE 4:**
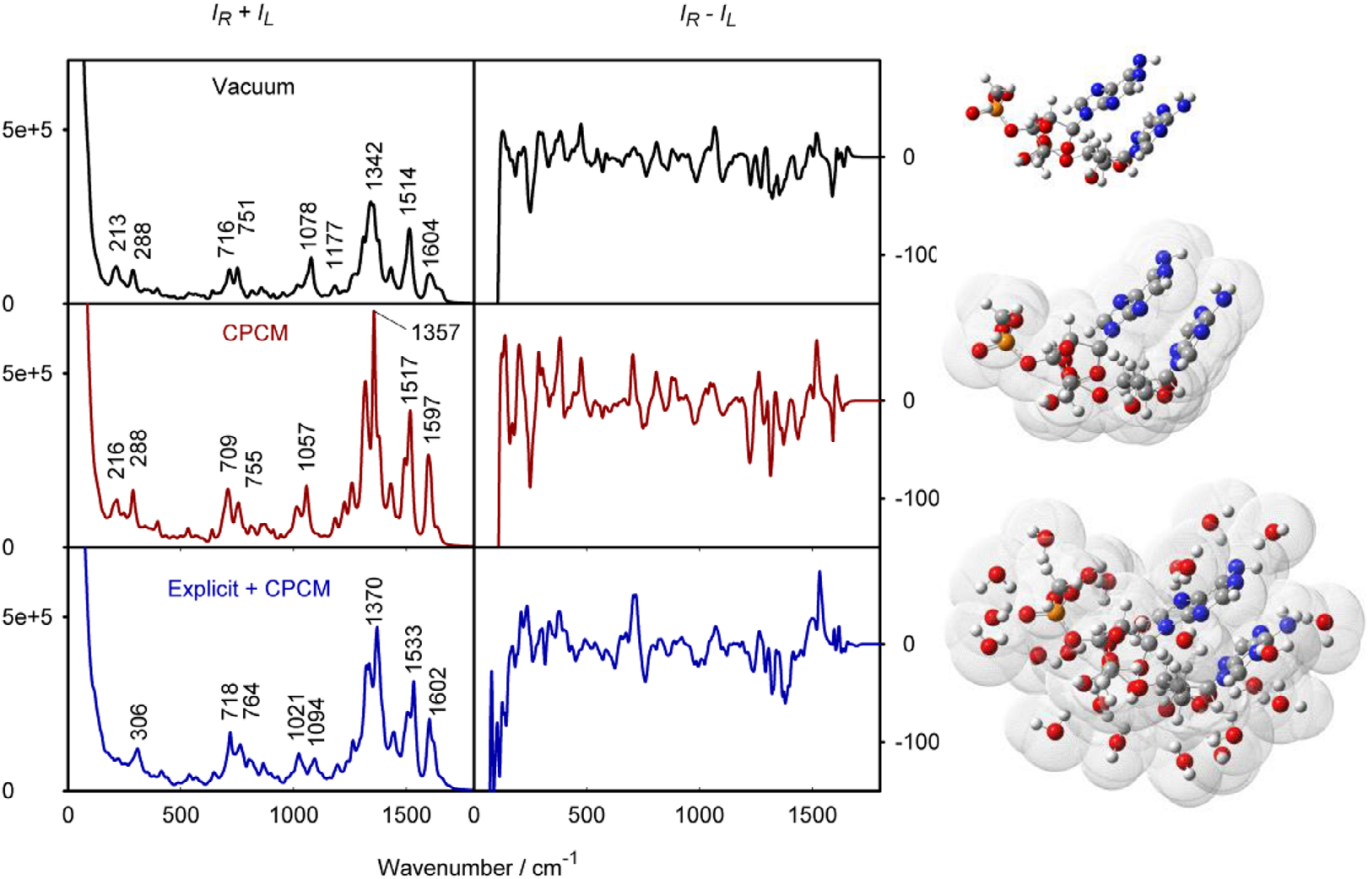
Raman and ROA spectra of the A_2_ model dimer in the standard A‐conformation, calculated with three solvent models: vacuum, the CPCM dielectric environment, and explicit H‐bonded water molecules combined with CPCM. The calculated spectra are obtained as averages from 10 snapshots, example geometries are plotted on the right.

The symmetric P═O (double bond) stretching is affected slightly more as it is moving from 1078 cm^−1^ in vacuum over 1057 cm^−1^ for CPCM to 1094 cm^−1^ for the combined environment. The last value is quite close to the experiment for polyA (at 1097 cm^−1^) and the other polynucleotides, which suggests that the explicit hydrogen bonding is needed for accurate modeling of this vibration. The antisymmetric P═O vibration gives lower Raman intensities than the symmetric one, occurs around 1220 cm^−1^, and is heavily mixed with C─H bending modes.

The explicit water is also needed to reproduce the experimental Raman band at 318 cm^−1^ (calculated at 306 cm^−1^ with the combined solvent), and some minor improvements can be found for the relative intensities of the 1480/1571 cm^−1^ bands. Another shortcoming of the vacuum computation is underestimation of the relative intensity around 1338 cm^−1^ (mostly CH bending). The vacuum Raman intensities also appear smaller on average if compared to those predicted with the solvent. A deeper analysis showed that this is mostly caused by the hydrophobic adenine residue, where atomic polarizability derivatives are predicted to be larger in the polar environment.

The computed ROA spectra of the dimer (Figure 4, right) must be compared to the polyA experiment with caution; nevertheless some benefits of the inclusion of explicit water are apparent as well. Clearly, the water is significantly contributing to the strong predominantly negative ROA pattern below 400 cm^−1^. Interestingly, even the sign pattern in the middle part of the spectrum is affected when a solvent model is added, such as the 709/766 cm^−1^ couplet. Compared to vacuum, the CPCM and explicit+CPCM approaches give rather similar spectra above 400 cm^−1^, which also explains their similar performance for the polyA polymer (Figure S4).

### 2.4 Fragment Length‐Dependence and Convergence With Respect to the Number of Snapshots

To optimize the computational protocol, we tested various lengths of the fragments used for the CCT‐based spectral simulations. It is known that vibrational spectra sense the secondary structure on a relatively short distance. For peptides, for example, 2‐3 aminoacid units may suffice to reproduce many spectral features of a long polymer [73], although longer‐range effects were noted for some regular systems, such as condensed DNA [74], polyproline [75], or amyloidal protein fibrils [76]. For polyA, spectra obtained using three and four mononucleotide fragments were very similar (Figure S5). The largest differences appeared in the low‐frequency region, similarly as observed previously for G‐quadruplexes [63]. For higher frequencies the CCT transfer appears rather redundant, as averaging of the spectra of individual fragments already provides similar results (Figure S6). The possibility to limit the fragment size is convenient, as the computer demands of DFT steeply grow with the molecular size. This relative rigidity of the polynucleotide helices and the G‐quadruplex geometry is favorable for the simulations. For polyA, for example, 5 and 20 snapshots provided practically the same spectra (Figure S7).

### 2.5 Temperature Dependence of the Spectra

The temperature variations carry information about MD and stability. Most spectra exhibit a sigmoidal dependence on the temperature, indicating a two‐state system [66]. For simplicity, we therefore refer to the temperature changes of the geometry as “melting”, although the actual events can be more complex. According to the spectra, the polynucleotides react to increasing temperature differently. While polyA and polyU melt easily, polyC and polyG are more stable.

Possible temperature degradation of the samples was controlled by a repeated measurement at room temperature after the heating. In addition, significant photochemical decomposition would lead to a dramatic increase of background fluorescence and thus be easily detectable. To prevent a biological degradation, the EDTA chelating agent was added, following previous RNA ROA studies [33, 34, 43]. Under these condition, we did not observe a significant sample change during the measurements.

Within 0°C–100°C, the biggest changes were observed for polyA. This is consistent with earlier studies indicating a melting of the helical structure at elevated temperatures [18]. In Figure 5, simulated Raman and ROA spectra at 1°C, 40°C, 80°C, and 180°C are compared to the experiment. The value of 180°C is added as a computational exercise. In ROA, a significant intensity loss at 80°C occurs, compared to the lower temperatures, whereas only minor changes are seen in Raman spectra. For example, the 1304 and 1508 cm^−^^1^ Raman bands become stronger and the 813 and 1483 cm^−^^1^ ones weaker at 80°C. The negative 1339 cm^−^^1^ ROA band loses about 50% of its intensity within 1°C–40°C and finally shrinks 10‐times at 80°C.

**FIGURE 5:**
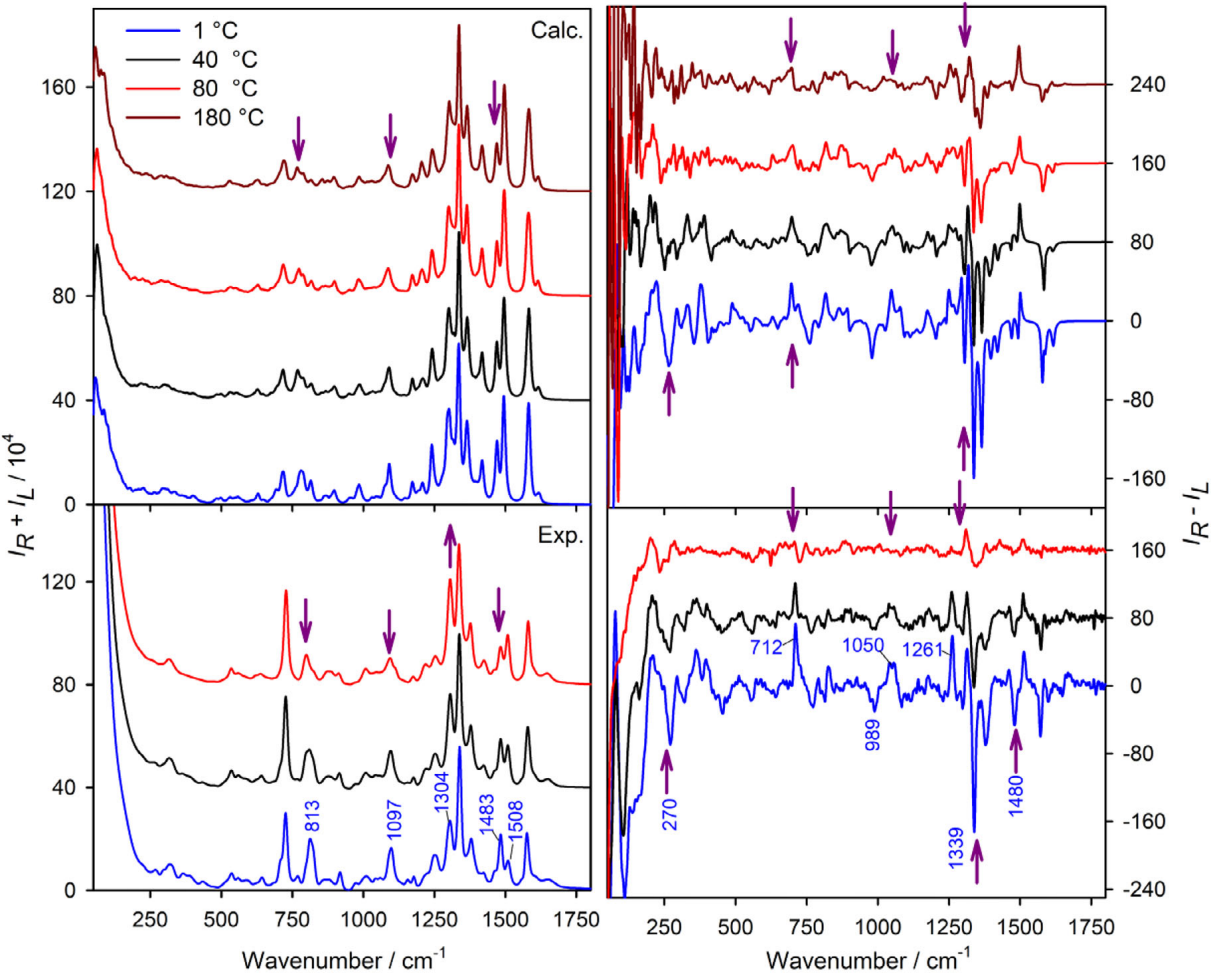
Calculated (top, CPCM solvation) and experimental (bottom) Raman and ROA spectra of polyA at different temperatures, the arrows indicate large intensity changes.

The simulations (Figure 5, top, selected spectral regions are compared in Figures S8 and S9) predict these trends; however, the changes in simulated intensities are more gradual and smaller than in the experiment. Even the 180°C simulation does not lead to the same extent of the ROA intensity loss observed at 80°C, which indicates limits in force field accuracy, most probably connected to a lack of polarization and cooperative effects. The RNA.Shaw force field may also be biased to reproduce a regular A‐RNA structure. The modest spectral variations in simulations correspond to gradual changes in the distributions of the internal coordinates (Figure 2). Apart from the force field limitations, changes of the acidobasic dissociation constants and consequent pH variations at elevated temperatures, not involved in the calculation, may also diminish accuracy of the simulations.

Compared to polyA, polyU melts at lower temperatures. Between 1 and 20°C its spectra change, while they are nearly constant within 20°C–80°C (Figure 6, top). Spectacular is the intensity loss of the negative ROA band at 1235 cm^−1^, quite strong at 1°C. For Raman, a slight decrease in intensity is observed for the bands at 786, 1095, and 1468 cm^−^^1^, whereas at 1685 cm^−1^ there is an intensity increase with temperature. The simulations in general reproduce this behavior; however, the predicted changes in ROA are more gradual and occur at higher temperatures.

**FIGURE 6:**
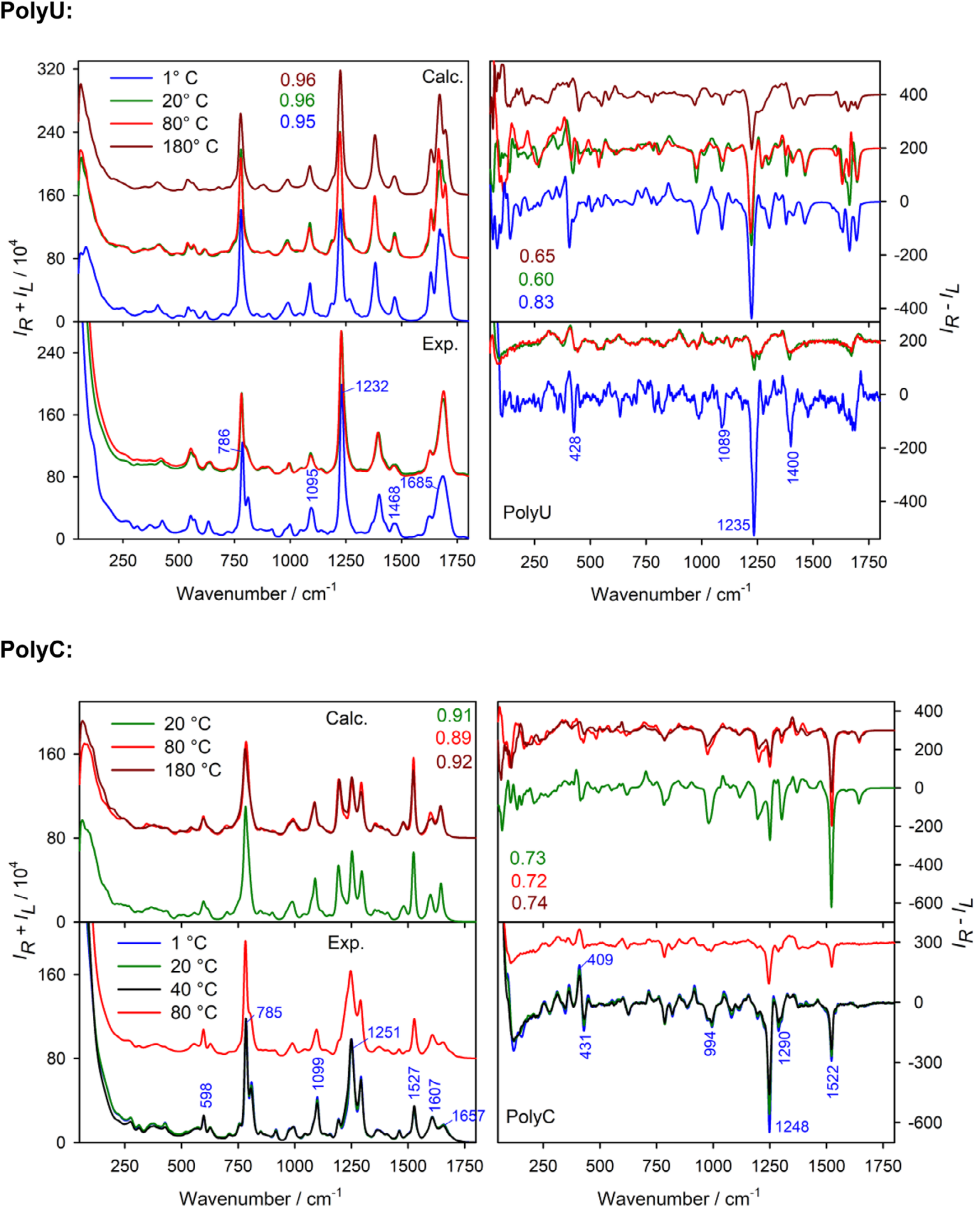
Calculated (upper panels) and experimental (lower panels) Raman and ROA spectra of polyU and polyC at different temperatures. The calculated spectra represent averages over 100 snapshots obtained with explicit solvation.

PolyC to some extent mimics polyA (Figure 6, bottom), with a similar transition temperature; only residual ROA intensity is larger at 80°C. This is consistent with the known behavior of polyC, supposed to adopt an ordered form at physiological pH [28, 77]. Again, the trends are reproduced by the calculations. Compared to polyA and polyU, relatively small changes occur in the lowest‐wavenumber (<300 cm^−1^) ROA. In all the polyA, polyU and polyC compounds CH and NH bending and base deformation modes appear as most sensitive to the temperature.

PolyG spectra do not change much with temperature (Figure 7), in line with the stability of the quadruplexes. The simulated Raman and ROA spectra reasonably well reproduced the experiment, except for higher wavenumber (> 1500 cm‐1) ROA, where the predicted strong bands are not realistic. Clearly, a better structural model is needed, which is, however, difficult for the tertiary structures as discussed elsewhere [63]. In addition, many conformations may be present in the solution, or the flexibility can be bigger than for our crystal‐based model.

**FIGURE 7:**
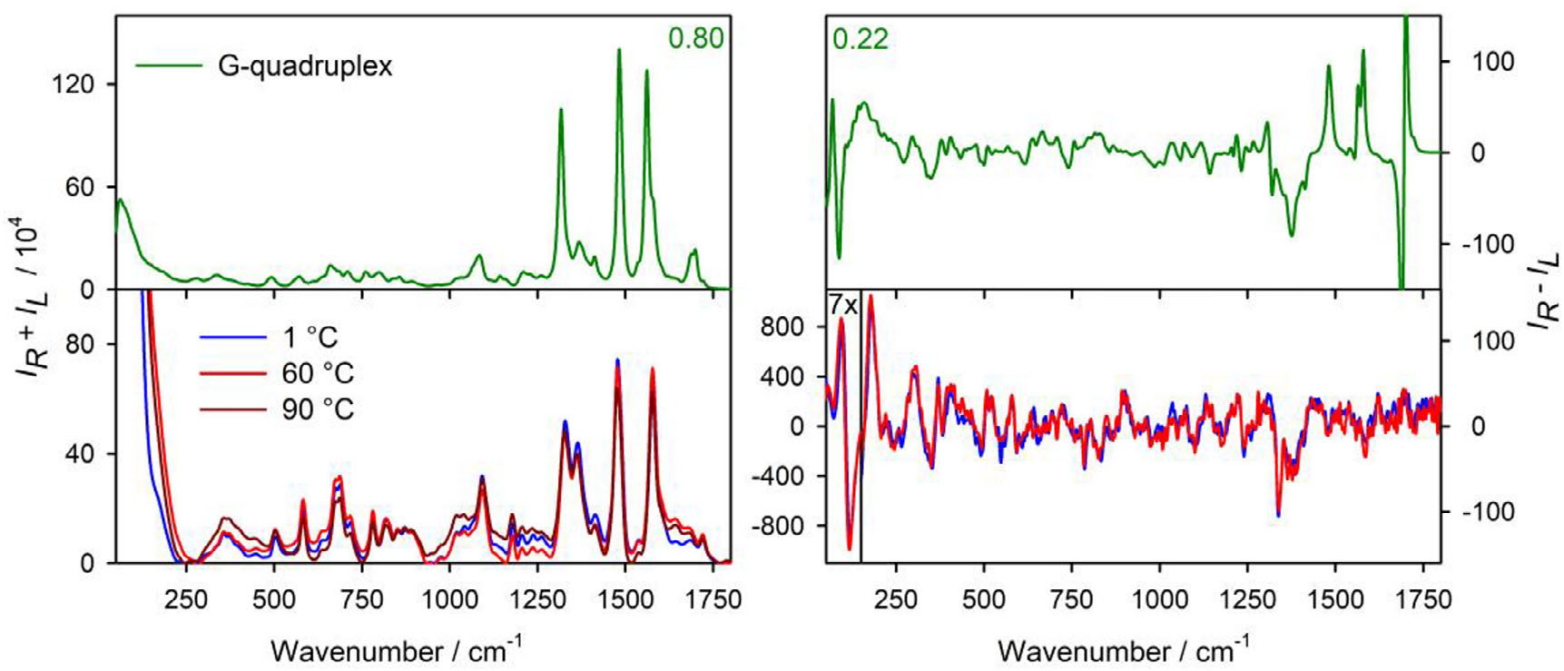
Raman and ROA spectra of polyG, calculation (top panels, at 20°C, for a quadruplex) and experiment (bottom, at 1°C and 60°C; at 90°C Raman spectrum was similar to that at 60°C, whereas the ROA was not measurable).

### 2.6 Conformational Sensitivity of ECD versus ROA

The conformational behavior found by ROA is consistent with the temperature dependence of UV absorption and ECD spectra shown in Figure 8. The ABS/ECD melting curves are also compared in Figure S13, melting temperatures are summarized in Table 2. Note that Raman spectra are less temperature dependent than ROA, and due to experimental noise and background signal in some cases Raman melting points cannot be reliably determined. Similarly as for ROA versus Raman, ECD is much more sensitive to the temperature variations than absorption.

**FIGURE 8:**
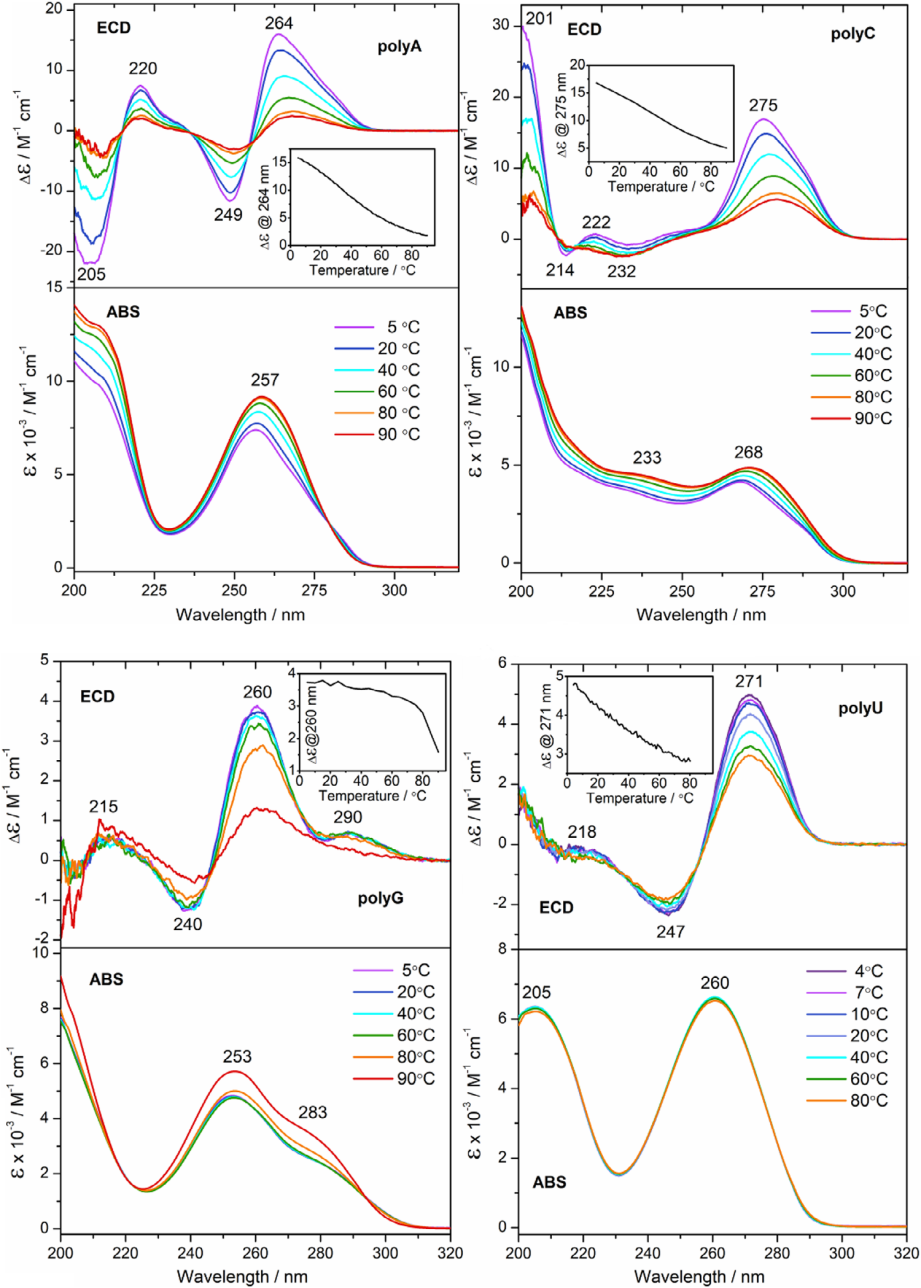
Experimental ECD (*Δε*) and UV absorption (*ε*) spectra of the studied polynucleotides. Melting profiles obtained from ECD spectra for selected wavelengths are inset.

**TABLE 2:** Melting temperatures (*T*_m_/°C) obtained by a fit of selected absorption, ECD, Raman, and ROA spectral intensities (for indicated wavelength or wavenumber).

	ABS	ECD	ROA	Raman
PolyA	34 (264 nm)	32 (264 nm)	57 (107 cm^−1^) 33 (1338 cm^−1^)	56 (1300 cm^−1^) 48 (1300 cm^−1^)^a^
			38 (711 cm^−1^)	
PolyU	5 (271 nm)	< 0 (271 nm)	< 0 (70 cm^−1^) < 0 (1235 cm^−1^)	< 0 (1100 cm^−1^)^a^
PolyC	38 (275 nm)	40 (275 nm)	42(107 cm^−1_^)	56 (1230 cm^−1^)
			64 (1245 cm^−1^) 84 (1522 cm^−1^)	49 (780 cm^−1^)
PolyG	>90 (260 nm)	89 (260 nm)	>90	

^a^ Background signal subtracted.

PolyA and polyC provide the most complete sigmoidal curves within 0‐100°C, their melting temperatures lie within 38–64°C. PolyA shows moderately cooperative transition of a stacked single‐stranded helix to an unordered coil‐like structure, which is confirmed by the presence of isosbestic points both in the UV absorption (∼280 nm) and in ECD (∼236 nm). On the other hand, polyC displays broader weakly cooperative transition with no clear isosbestic points in the spectra, suggesting a more gradual unstacking of the bases with temperature.

The regular polyU structure seems to be partially stable only close to 0°C. A small noncooperative decrease of ECD intensity and virtually no changes in the UV absorption spectra suggests only minimal local ordering at low temperatures. According to ECD, the ordered stacked structure of polyG breaks down at temperatures above 80°C, which follows from the highly cooperative collapse of ECD intensity. This was not observed in Raman/ROA where higher concentrations were used, stabilizing the ordered form. Unfortunately, the same concentration cannot be used in all the techniques due to restrictions related to different sensitivity, absorption, and sample manipulation. For ROA, the spectra cannot be accumulated at too many temperature points due to the long accumulation times; nevertheless, some transitions appear sharper than in ECD (Figure S10), depending on the chosen wavenumber. The melting temperatures can differ depending on the chosen spectral range: different parts of ROA spectra, similarly as for ECD, may reflect different processes in the sample. In particular, low‐frequency (< 200 cm^−1^) ROA reflects not only polynucleotide conformation but also changes in the water properties and its interaction with the biomolecules.

### 2.7 Marker Bands of Nucleotide Polymerization

Apart from the conformational sensitivity, the spectra may also be used to track nucleotide polymerization. This is shown by comparing Raman and ROA spectra of polymeric G, C, U, and A to corresponding monomers (Figure 9). The monomer spectra are described in detail in a previous study [35]. In Raman, polymerization “marker bands” reflect the creation of the covalent bonds in the polymers. They namely include the phosphate group vibrations within 750 and 1150 cm^−^^1^. The intense Raman band at ∼980 cm^−^^1^ characteristic for the terminal PO_3_^2^^−^ symmetric stretch splits to bands at ∼815 and ∼1100 cm^−^^1^ corresponding to the symmetric P─O and P═O stretching vibrations of the phosphate groups in the polymers. Previously, the intensity ratio at 815 and 1100 cm^−1^ was related to the conformation; values around 1.64 indicated ordered RNA structures, smaller ones were assigned to disordered systems [78]. For polyG, we can see a weak Raman band (∼1721 cm^−^^1^) and a clear ROA couplet (∼1698 cm^−^^1^) of the carbonyl stretching absent in the monomer and characteristic for the quadruplex forms [63].

**FIGURE 9:**
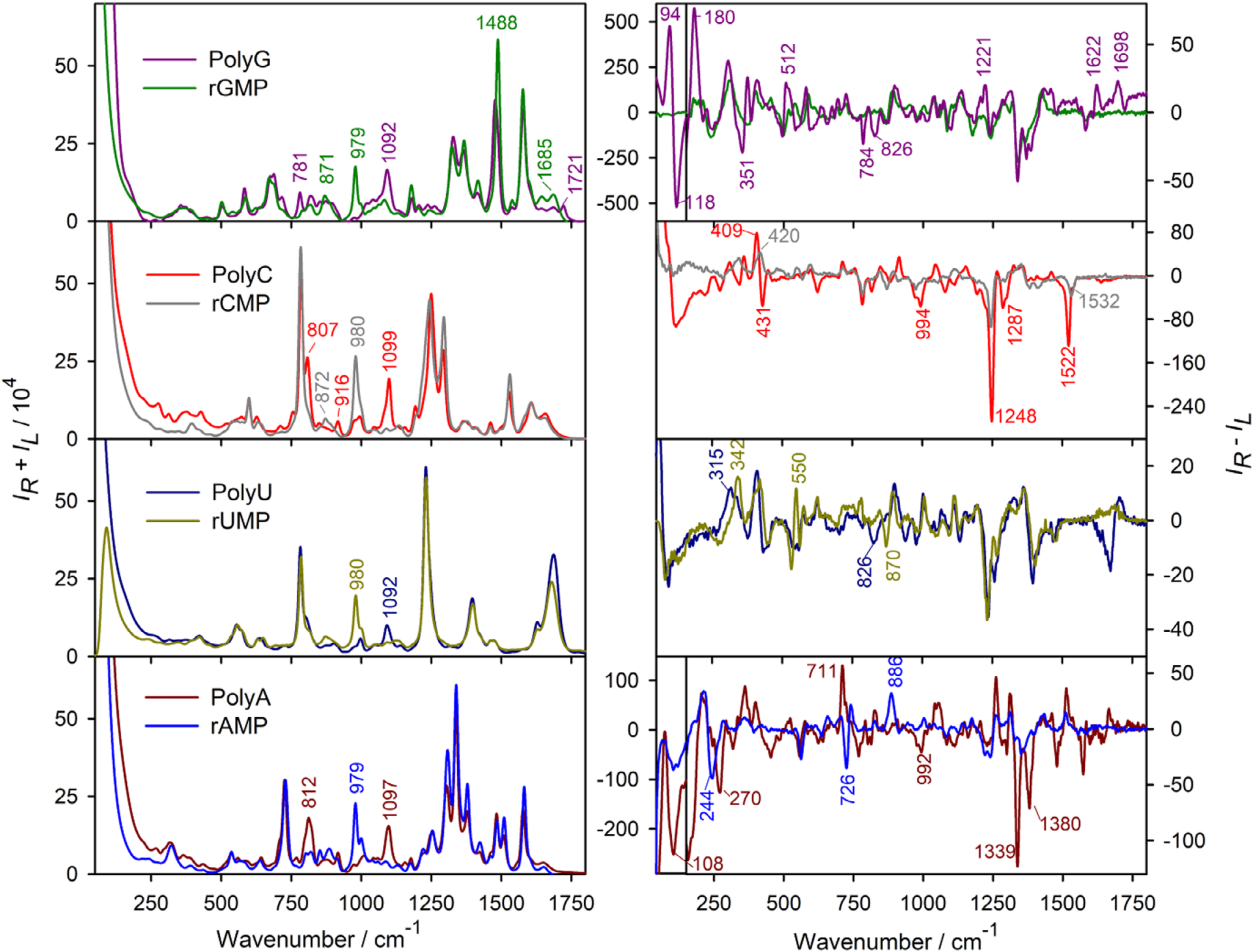
Experimental Raman and ROA spectra of the monomers and polymers of A, U, C, and G. Raman and ROA intensities are normalized to Raman areas. Note the different intensity scales at low (<200 cm^−1^) and high frequencies in the first and last ROA panels.

In higher‐frequency (>250 cm^−1^) ROA spectra, the helical polyC and polyA compounds provide enhancement of several bands. This can be related to longer‐range mode coupling, similarly as found for polyproline, for example [79]. Interestingly, the 711 cm^−1^ band, which arises from a combination of base‐breathing and sugar vibrational modes, changes sign in monomeric and polymeric adenine. An even more convincing indicator is the region below 250 cm^−1^, dominated by the delocalized skeletal modes, similarly as in proteins [53, 80]. The lower ROA intensity here for polyC may mean a higher degree of conformational disorder in this polymer compared to polyA and polyG.

### 2.8 The CH Stretching Region

Although the CH stretching ROA is measurable (around 2900 cm^−1^, Figure S11), unlike for the opposite lowest‐frequency region, a high noise‐to‐signal ratio makes it less useful for structural monitoring compared to the mid region (∼500‐ 1800 cm^−1^). All nucleotides exhibit predominantly positive ROA in the 2900–3000 cm^−1^ range of aliphatic C─H vibrations, while the ROA signal of aromatic CH stretching (around 3100 cm^−1^) is to weak to be detectable. Both Raman and ROA signals are unusable above 3100 cm^−1^, where it is difficult to subtract water OH stretching signal and associated artifacts [81]. The computations (Figure S12) do reproduce the overall positive CH stretching ROA, albeit at higher frequencies than in experiment because of the anharmonic effects [72]. Polynucleotide NH and OH stretching bands (above ∼3100 cm^−1^) are not resolved in experimental spectra because of the broadening caused by hydrogen bonding and the strong signal from water.

## 3 Conclusion

To advance the methodology and obtain insight into the molecular behavior, we measured Raman and ROA spectra of four model polynucleotides in a wide wavenumber region and for several temperatures. A combined MD/DFT computational protocol was developed and tested, which with a high degree of fidelity reproduced the experimental trends, providing a link between molecular geometry, dynamics, and the spectral shapes.

The results show that based on the comparison of computational and experimental data, Raman and ROA spectroscopy can be used to validate MD force fields for nucleic acid systems in solution, identify marker bands characteristic for nucleotide polymerization, and identify parameters needed for faithful spectral simulations, such as the solvent model and fragmentation strategy. The temperature dependence of the vibrational spectra was consistent with the data from UV absorption and ECD measurements, although some spectral regions exhibited a more complex behavior, such as reflecting temperature properties of the solvent and its interactions with the polynucleotides.

Both calculations on the dinucleotide model and polyA indicated that while Raman intensities can be relatively reliably simulated in vacuum, more faithful solvent model, preferably explicitly including some water molecules, should be used for simulation of ROA.

To better describe the behavior of these molecules, many challenges for the theory remain, such as more accurate MD force fields and more appropriate solvent models for DFT. The current and future improvements of the spectroscopic and computational methodologies will enable the development of practical means universally usable for nucleic acid solution studies.

## Conflicts of Interest

J. Kap. is involved in commercialization of an ROA spectrometer, the other authors declare no conflict of interest.

## Supporting information



Details of experimental and computational methodology, computational tests, and supporting experimental data. The authors have cited additional references within the Supporting Information [82, 83, 84, 85, 86, 87, 88].
**Supporting File 1**: chem71445‐sup‐0001‐SuppMat.pdf.

## Data Availability

The data that supports the findings of this study are available in the Supporting Information of this article.
