# Supplementary material for: Raman Optical Activity as a Tool to Monitor Polynucleotide Conformation in Solutions
Source: Chemistry. 2026 Jul 29;32(40):e71445. doi: 10.1002/chem.71445 (PMC13716028; doi:10.1002/chem.71445)
Supplement: Supplementary file 1 [file CHEM-32-e71445-s001.pdf]

Supporting Information

Raman Optical Activity as a Tool to Monitor Polynucleotide Conformation in Solutions

Mohammed Siddhique Para Kkadan, Josef Kapitán,[c] Jiří Kessler,
Ondřej Zapletal, Valery Andrushchenko, and Petr Bouř

Contents

Methods
Figure S1. Hydrated A_{11} and example of trinucleotide fragments surrounded by water molecules.
Figure S2. Temperature dependence of the sugar pucker and χ distributions, as obtained from MD.
Figure S3. Probability distributions of P and χ for the RNA.OL3 and RNA.Shaw force fields.
Figure S4. PolyA, Raman and ROA spectra calculated with the implicit and combined solvation,
Figure S5. Raman and ROA polyA spectra simulated with three and four mononucleotide units.
Figure S6. Raman and ROA polyA spectra calculated as averages of individual fragments vs. full CCT.
Figure S7. Raman and ROA polyA spectra of simulated with different numbers of snapshots.
Figure S8. PolyA and PolyU, details of Raman and ROA spectra for several temperatures.
Figure S9. PolyA, differential Raman and ROA spectra ("80°C minus 1°C").
Figure S10. Dependence of selected ROA and Raman spectral intensities on temperature.
Figure S11. Experimental Raman and ROA spectra of the polynucleotides, full wavenumber range.
Figure S12. Calculated Raman and ROA spectra of the polynucleotides, full wavenumber range.
Figure S13. UV absorption ECD melting curves.

Table S1. Experimental and calculated frequencies of selected Raman and ROA bands.
Table S2. Calculated P-O and P=O stretching frequencies of dimethylphosphate.

Methods

Spectra Measurement

PolyA, polyC, polyG, and polyU potassium salts were purchased from Sigma-Aldrich and dissolved in Tris (10 mM)/EDTA(1 mM) buffer (pH 7.4 at 25°C) at concentrations of 25 (polyA) and 50 (the rest) mg/mL for Raman and ROA experiments. The spectra were recorded with a spectrometer located at the Palacký University in Olomouc,[1] operating within 50-4500 cm^{-1}, in the scattered circular polarization (SCP) modulation, backscattering geometry, and using 532 nm laser excitation. The samples were held in a fused silica cell of ~70 μl volume, the temperature was stabilized by a thermostat, laser power at the sample was 800 mW, the spectra were accumulated for about 30 h. Water signal was subtracted from Raman spectra, and minor baseline correction made.

As an alternative method to monitor temperature-dependent conformational changes, UV absorption and electronic circular dichroism (ECD) spectra were obtained at several temperatures, for polynucleotide concentrations of 0.5 mg/mL in Tris (10 mM)/EDTA(1 mM) buffer (pH 7.4 at 25°C), with a Jasco J-815 spectrometer using 1 mm pathlength and 10 nm/min scanning speed.

From selected points of absorption, ECD, Raman and ROA spectra recorded for different temperatures "melting temperatures" T_m were obtained by a fit, using a Boltzmann function,[2]

$$S(T) = S_{\max} + \frac{S_{\min} - S_{\max}}{1 + e^{(T - T_m)/d}},$$

where S_{min} and S_{max} are limitied values for low and high temperatures, respectively, and d is a parameter.

Molecular Dynamics

Initial geometries of all polynucleotides were obtained from the Protein Data Bank (PDB, https://www.rcsb.org), containing 11 (polyA) or 16 (the rest) residues. Standard A-DNA torsion angles were used for initial geometry, except for polyG adopting a G-quadruplex.[3, 4] Thus a PDB G-quadruplex structure 2MB2[5] was taken for polyG.

Each molecule was then put in a cubic box (70 Å)^3 otherwise filled with water and potassium counterions, using the Packmol software.[6] After minimization and a short equilibration, *NVT* simulations at selected temperatures were run for 500 ns. The Langevin thermostat[7] with a collision frequency of 2 ps^{-1} was used, non-bonded interactions were cut-off at 8 Å, and the trajectory was analyzed using the Cpptraj module within AmberTools.[8]

Simulation of Vibrational Spectra

20 snapshot geometries were extracted at regular 10 ns intervals, and only waters of the first solvation shell closer than 3 Å were kept. For computational tests, all waters were deleted. Using an automatic fragmentation procedure, similar to that used for peptides,[9] the snapshot geometries were divided into overlapping trinucleotide units including the surrounding solvent molecules. An example of the fragmentation is given in **Figure S1**.

Quantum chemical calculations on the fragments were carried out using the Gaussian 16 software package.[10] The geometries were partially optimized in the normal mode coordinates[11, 12] where vibrational modes with wavenumbers below 100 cm^{-1} were constrained. The B3LYP[13] functional with the D3 dispersion correction[14] and the 6-31++G** (6-31G** for the solvent) basis set were used. Water molecules not included explicitly were accounted for by the conductor polarizable continuum model (CPCM).[15] The force field and polarizability derivatives[16] were calculated at the same level as the optimized geometries and transferred back on the original geometry, using the Cartesian coordinate tensor transfer (CCT).[17-19] Raman and ROA intensities were then computed at the harmonic level and averaged over the snapshots. Since the calculated P=O stretching frequency was anomalously low, the P=O bond force constant was scaled separately (cf. ref. [20]). For a better comparison with the experiment in the figures, the calculated frequencies were multiplied by 0.98.

Since it is not possible to measure absolute Raman intensities, the simulated Raman and ROA intensities were multiplied by the same factor, making calculated and experimental Raman areas within 450-1800 cm^{-1} the same. For a fast comparison of the simulated and experimental spectra, similarities were calculated as $s = \int S_{sim} S_{\exp} d\nu / \sqrt{\int S_{sim}^2 d\nu \int S_{\exp}^2 d\nu}$, where the calculated frequencies were multiplied by a factor giving the highest *s*. The integrations ran within 450-1800 cm^{-1}.

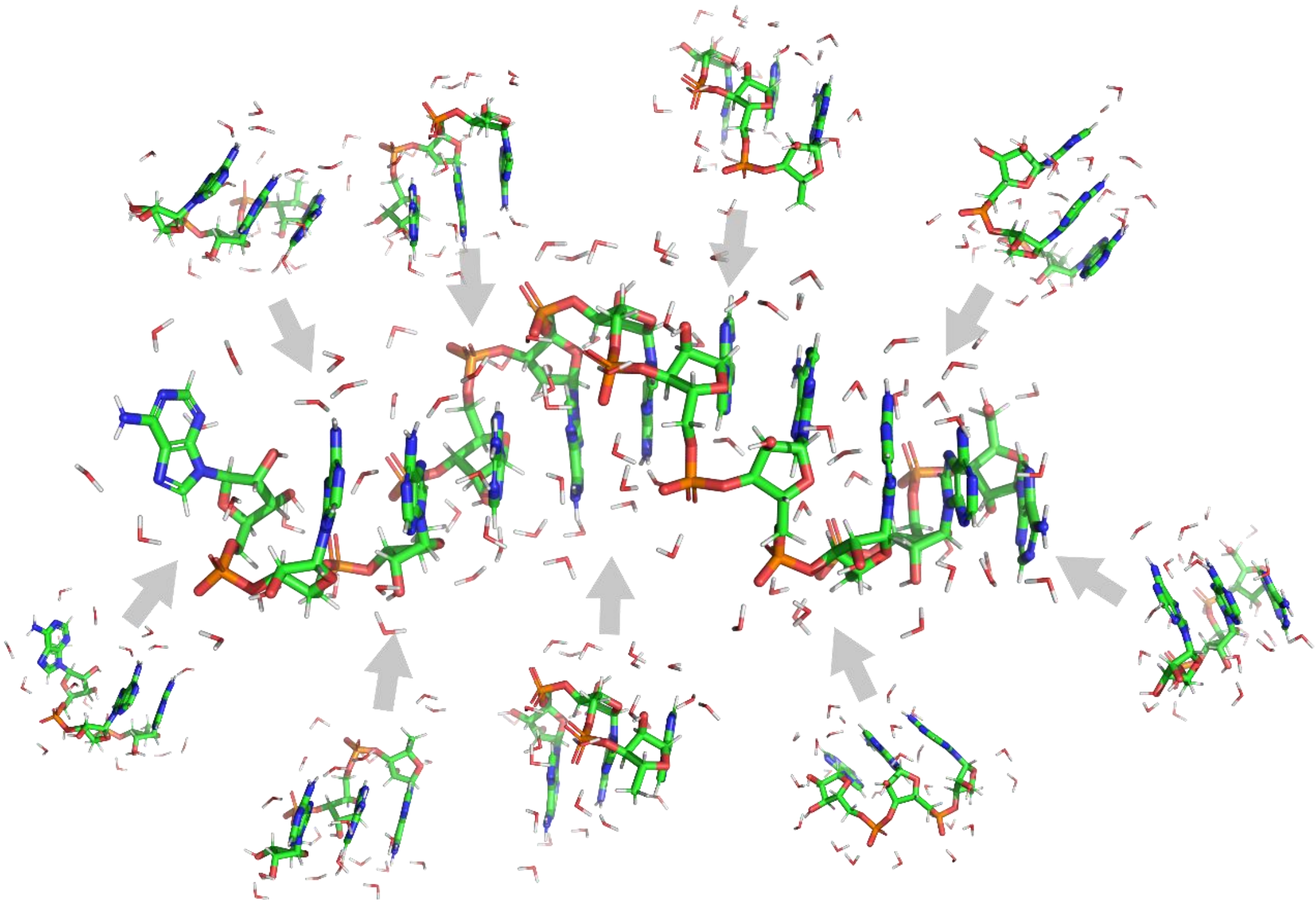

Figure S1. Hydrated A_{11} (center, example snapshot) and trinucleotide fragments surrounded by water molecules used as sources of the vibrational parameters. These were transferred on the snapshot, so that its vibrational spectra could be generated.

PolyU

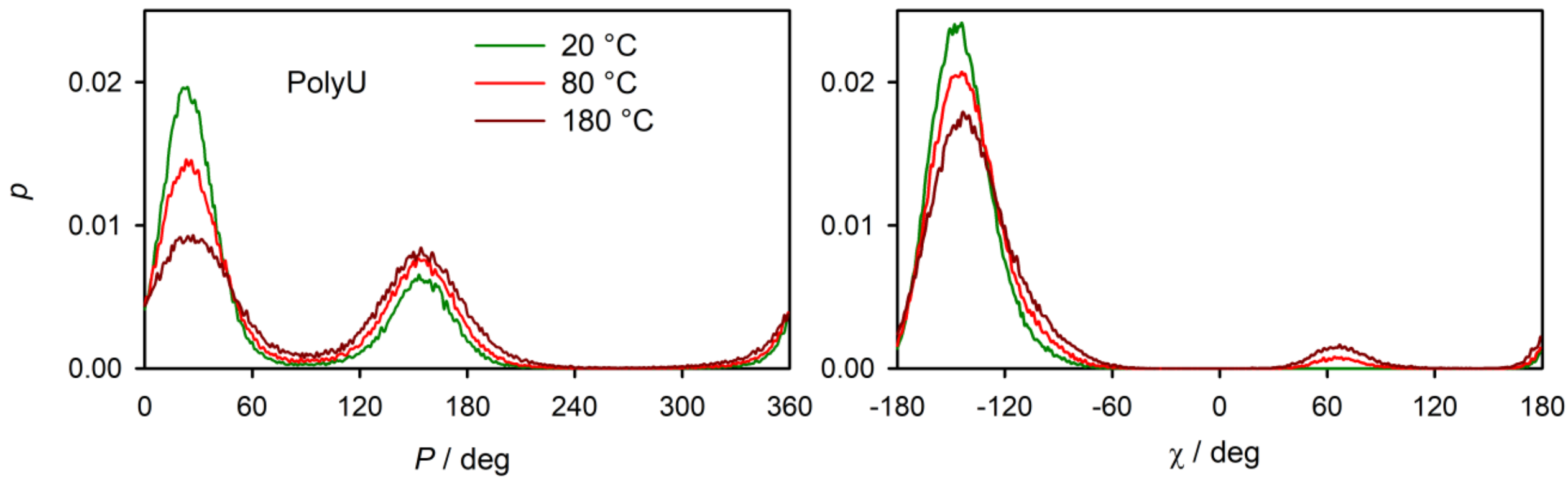

PolyC

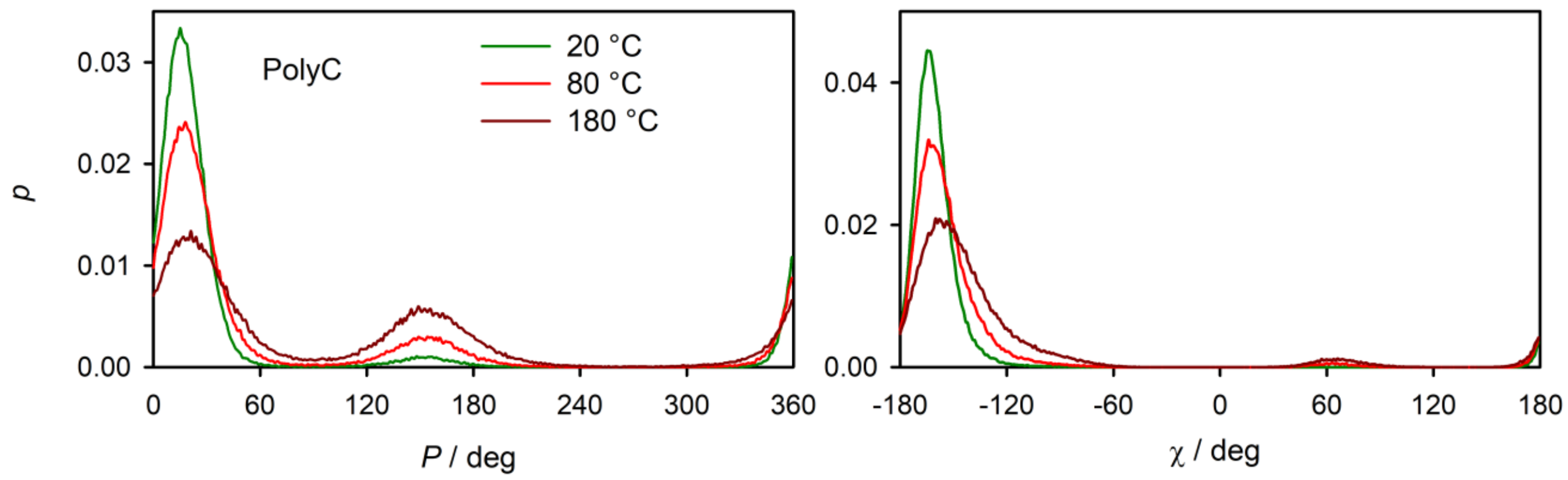

Figure S2. Temperature dependence of the sugar pucker (*P*) and glycosidic torsion angle (χ) distributions, as obtained from MD, for polyU and polyC.

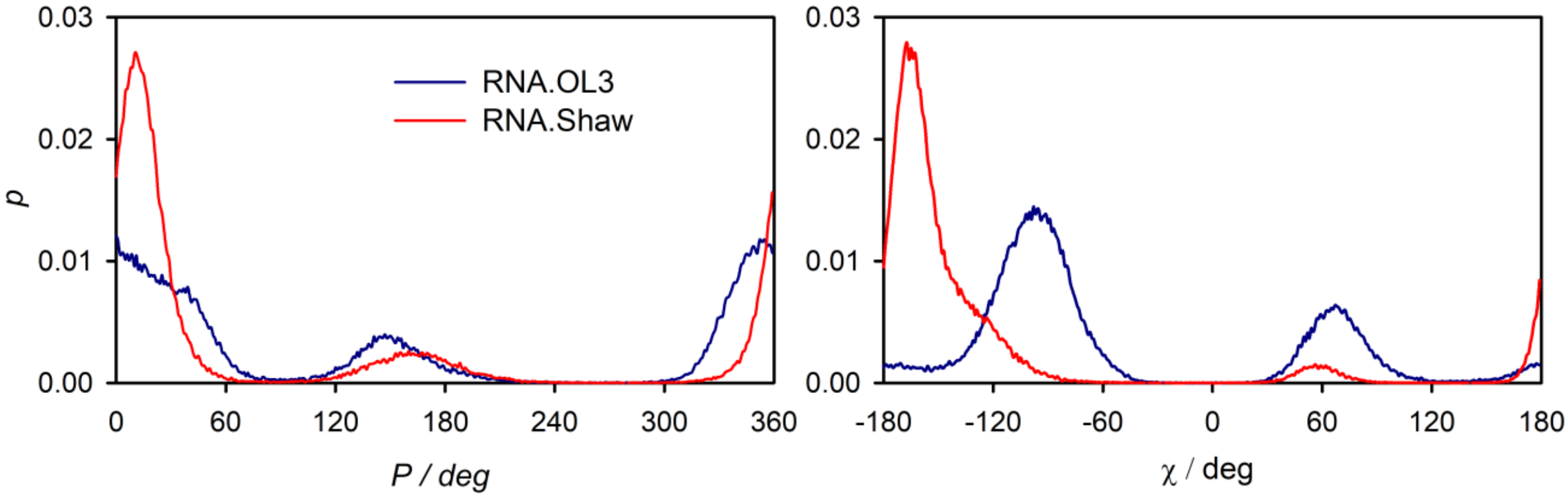

Figure S3. Probability distributions of the sugar pucker (*P*) and glycosidic torsion angle (*χ*) as obtained from MD for polyA using the RNA.OL3 and RNA.Shaw force fields.

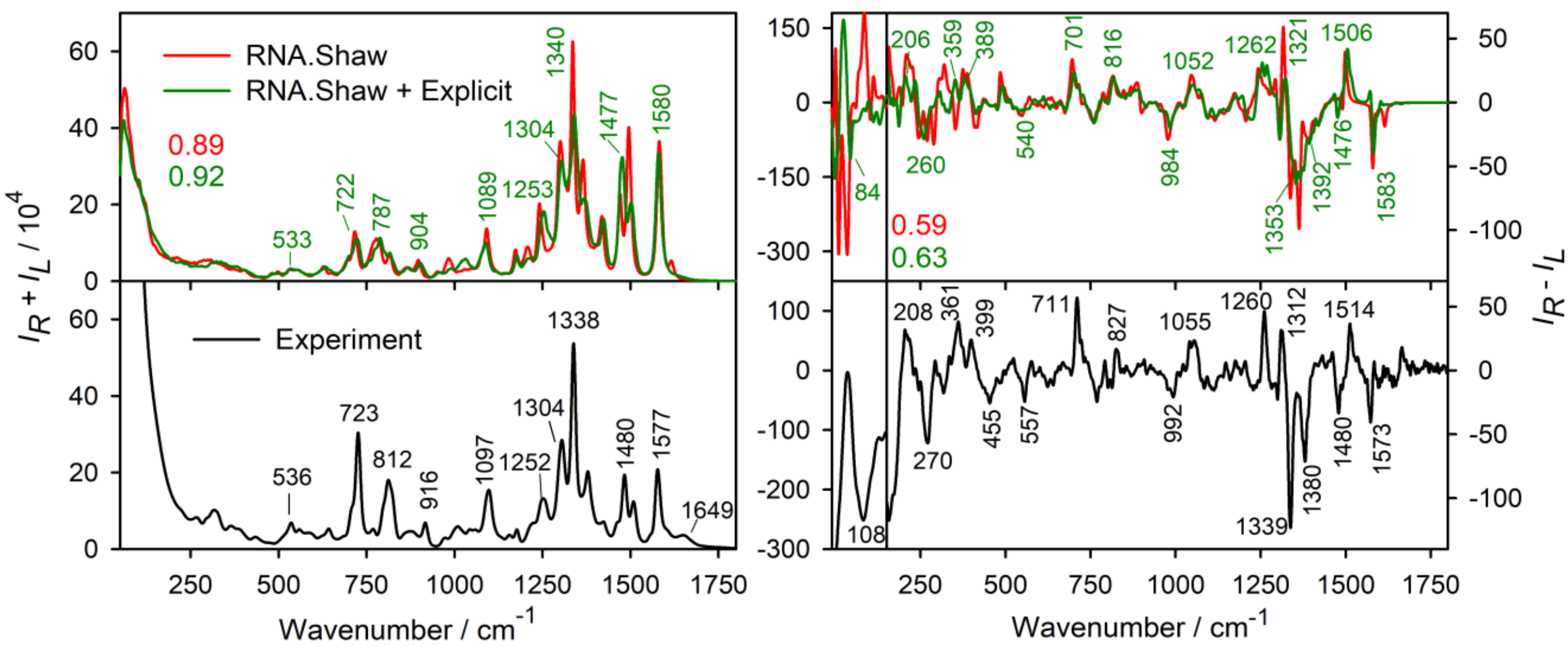

Figure S4. Raman and ROA spectra of polyA calculated with the CPCM and combined explicit+CPCM solvation. ROA spectra below and above 200 cm^{-1} are shown with separate scales indicated on the left and right axes, the red and green numbers correspond to similarities of the calculated spectra to the experiment.

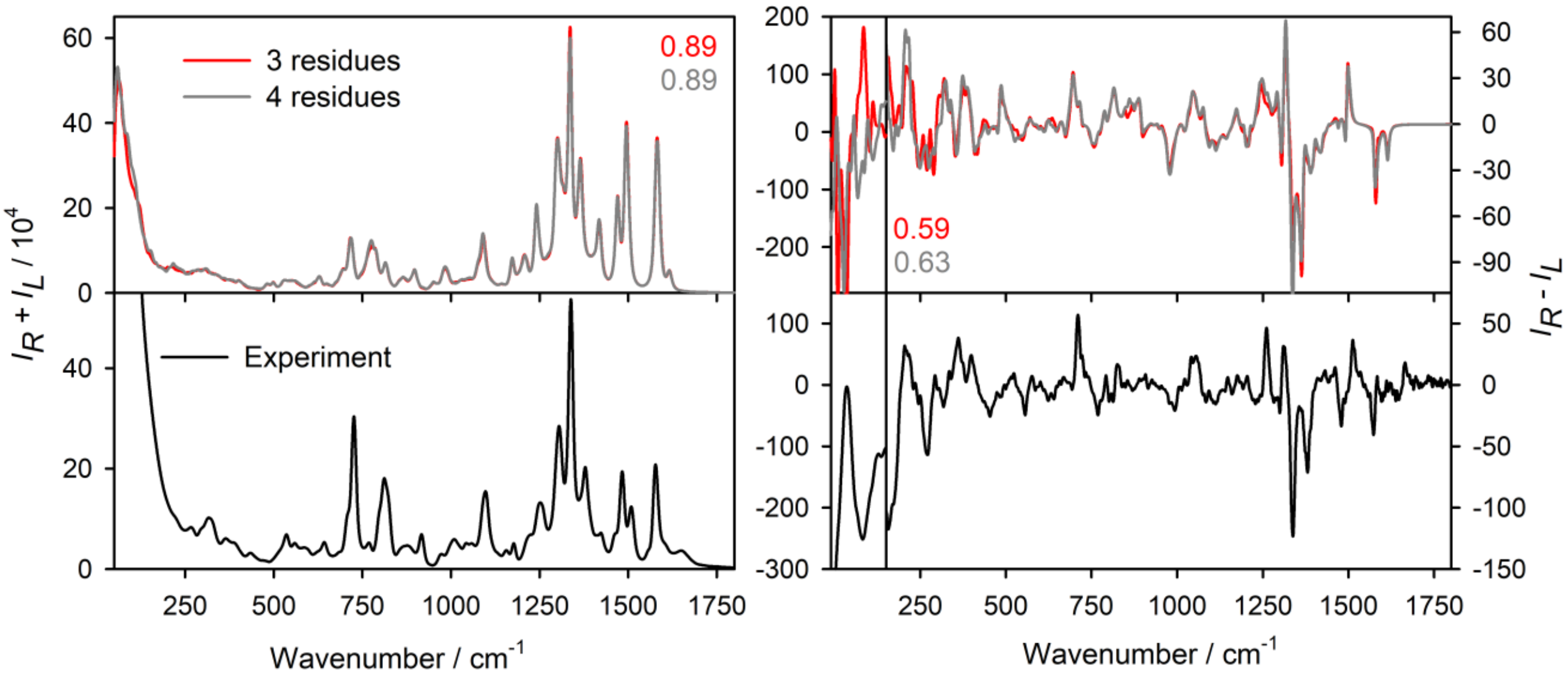

[**Figure S5.**]() Raman and ROA polyA spectra of polyA simulated with three (red) and four (green) mononucleotide units, using combined explicit+CPCM solvation, the red and green numbers show similarities of the calculated spectra to the experiment.

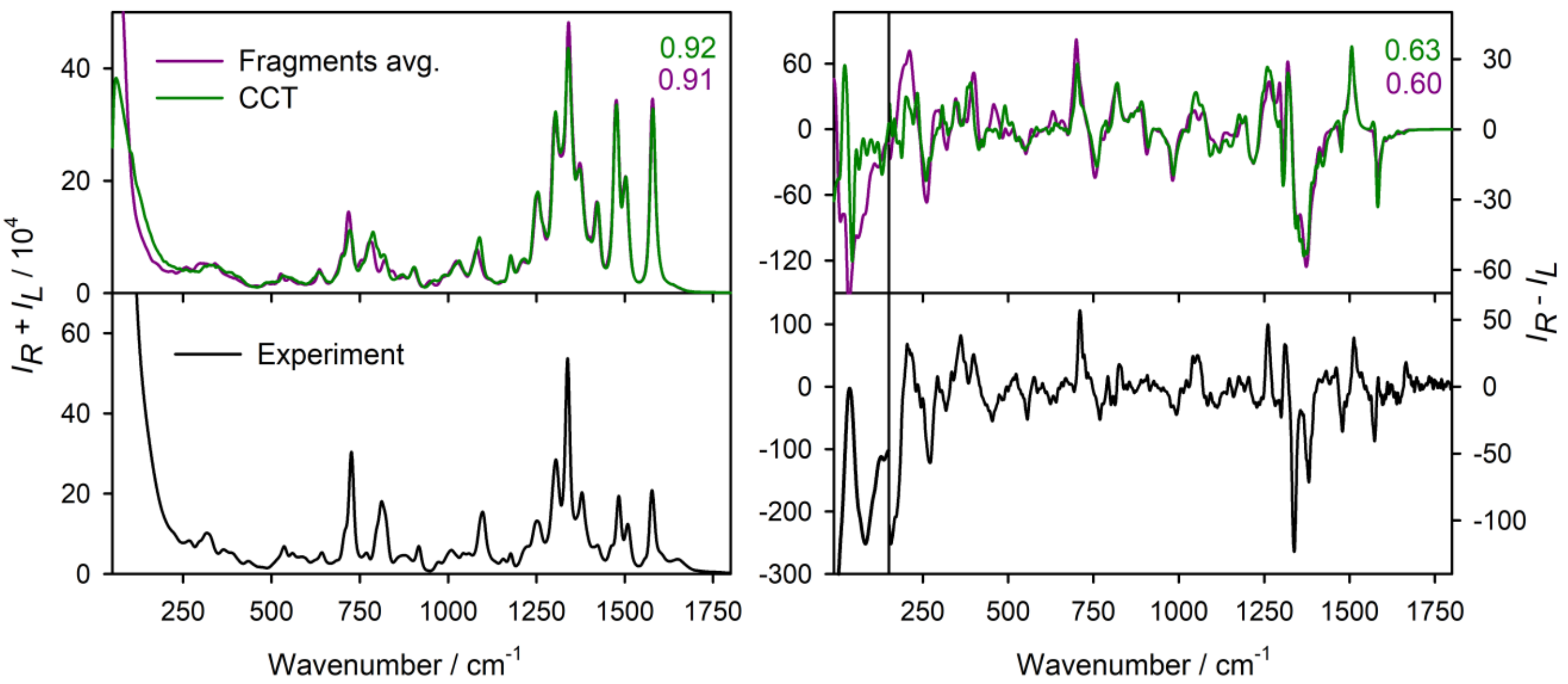

[**Figure S6.**]() Raman and ROA polyA spectra calculated as averages of individual fragments vs. full CCT calculation (average over 20 MD snapshots, explicit+CPCM hydration). The blue and red numbers indicate similarities of the calculated spectra to the experiment.

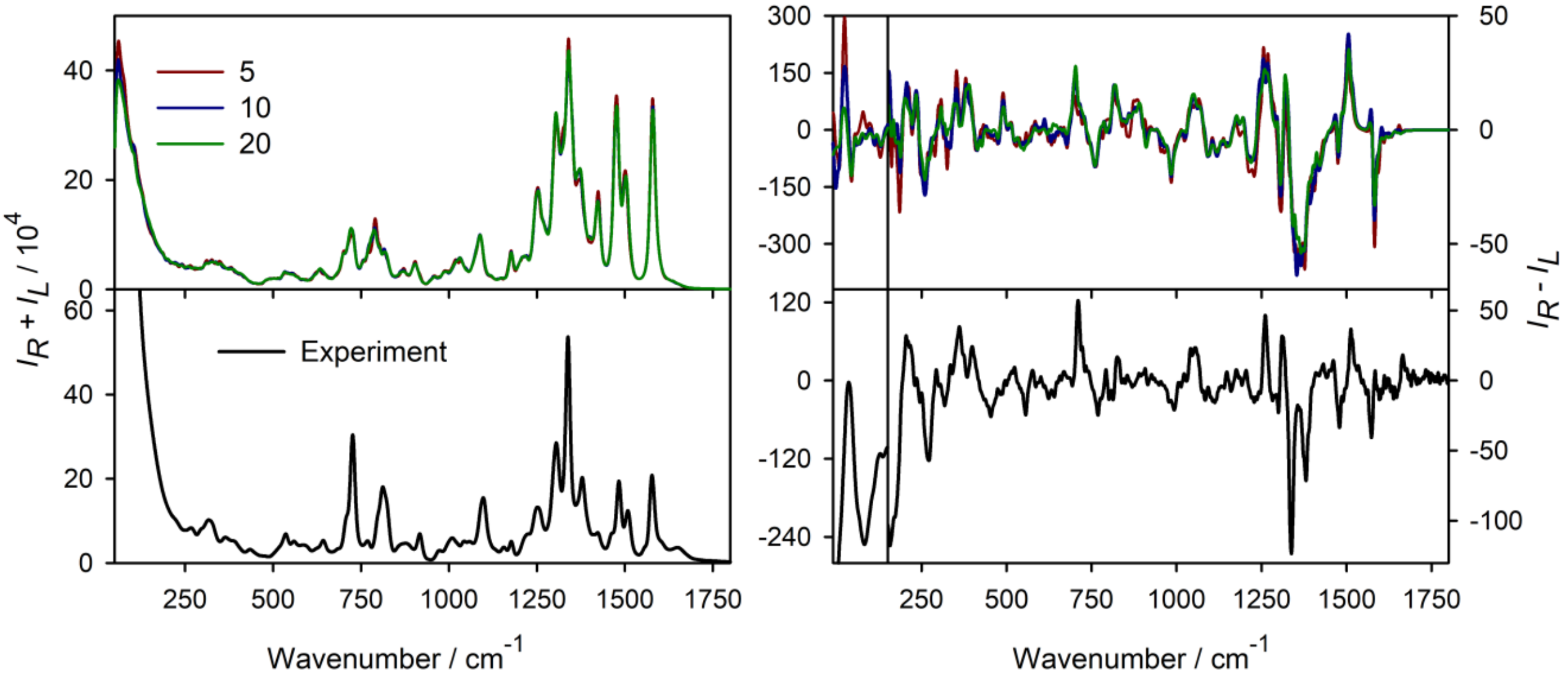

Figure S7. Raman and ROA polyA spectra of simulated as averages of 5 (red), 10 (blue), and 20 (green) snapshots. For explicit+CPCM solvation, experiment at 20 °C.

PolyA Raman:

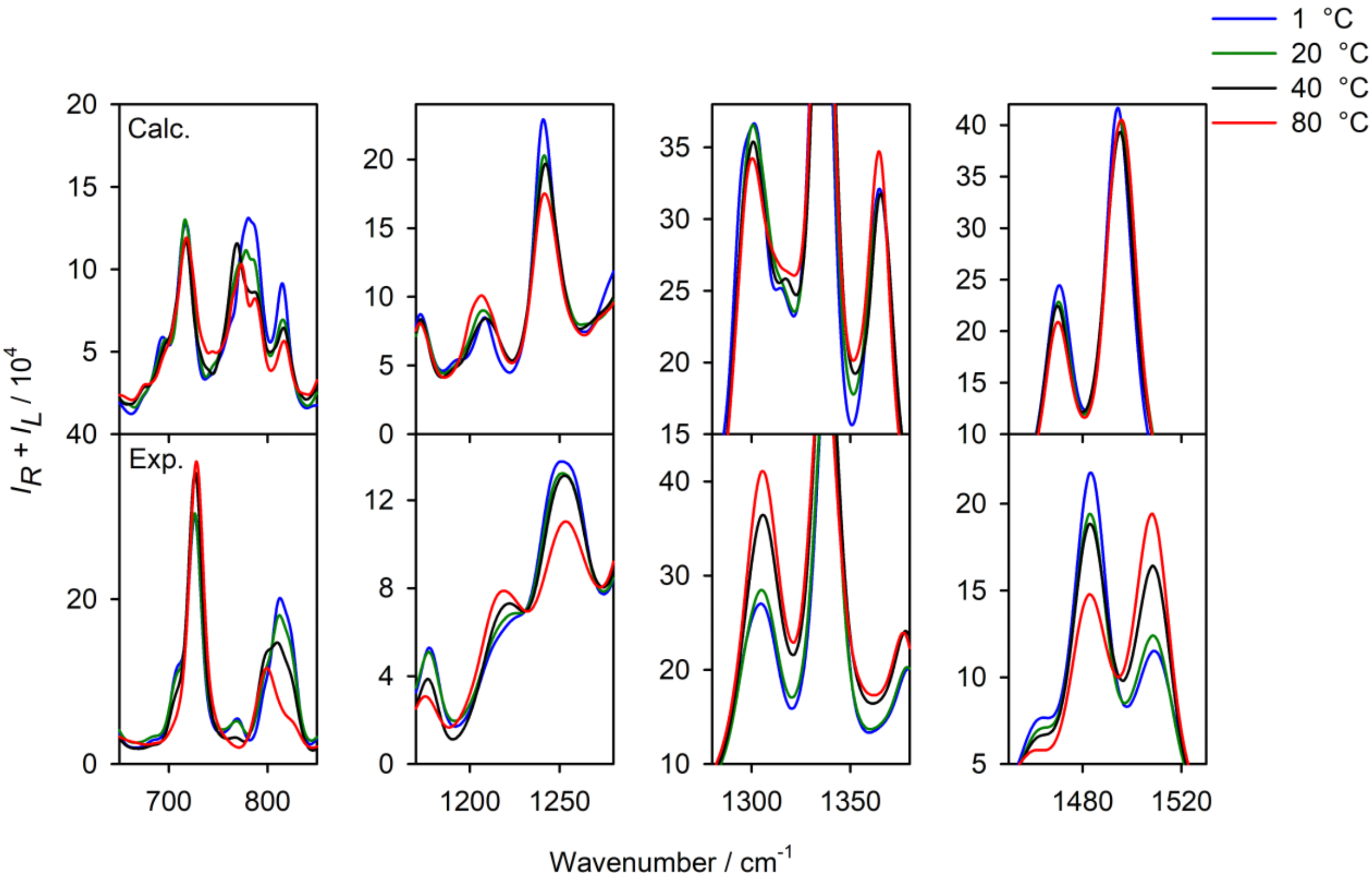

PolyA ROA:

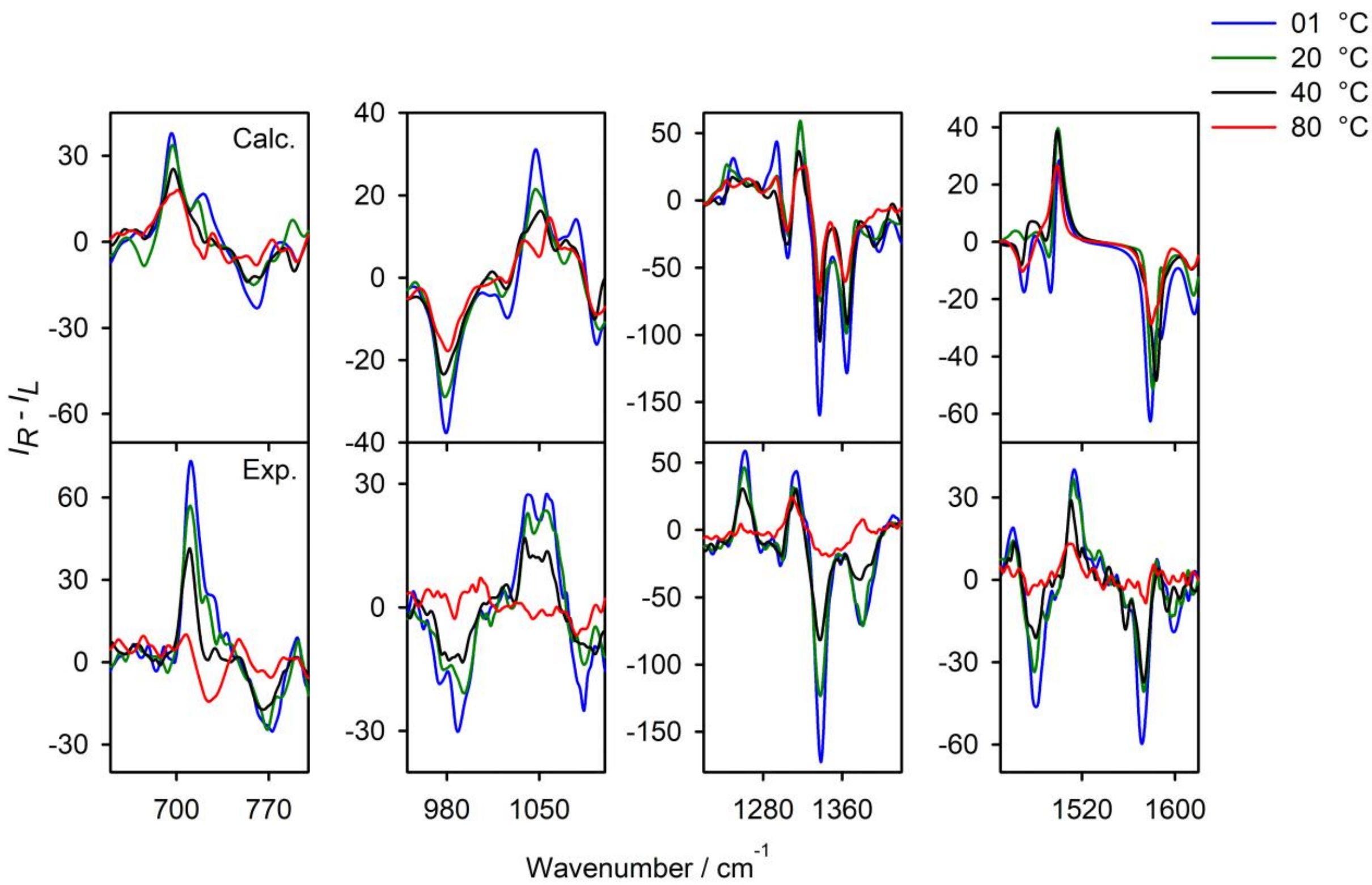

PolyU, Raman:

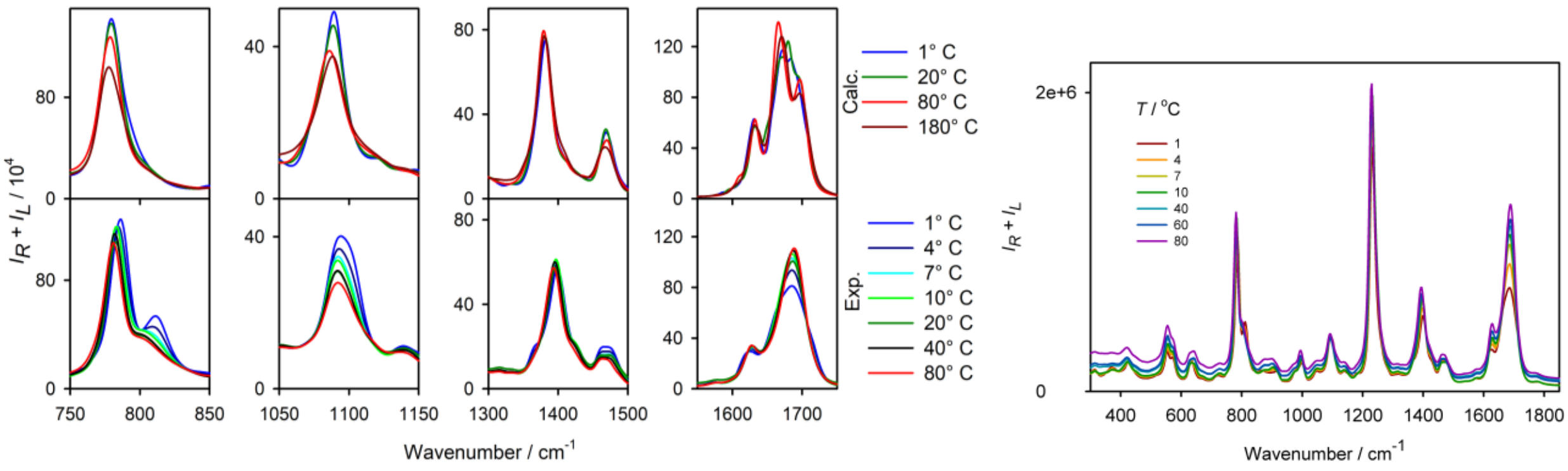

PolyU, ROA:

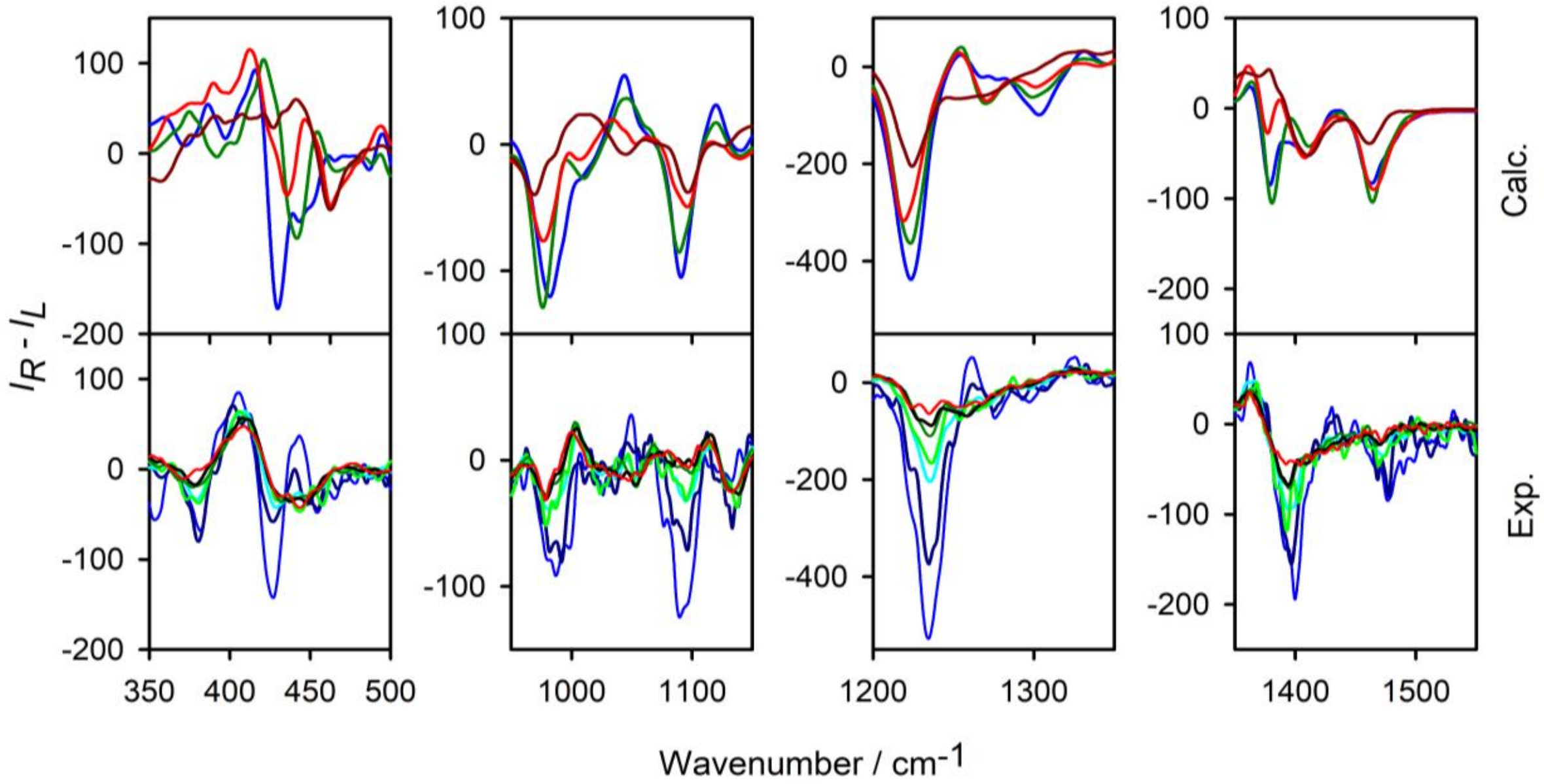

Figure S8. PolyA and PolyU, simulated (top panels) and experimental (bottom) Raman and ROA spectra for several temperatures in selected wavenumber regions. Raman spectra are shown with (left) and without (right) baseline subtraction.

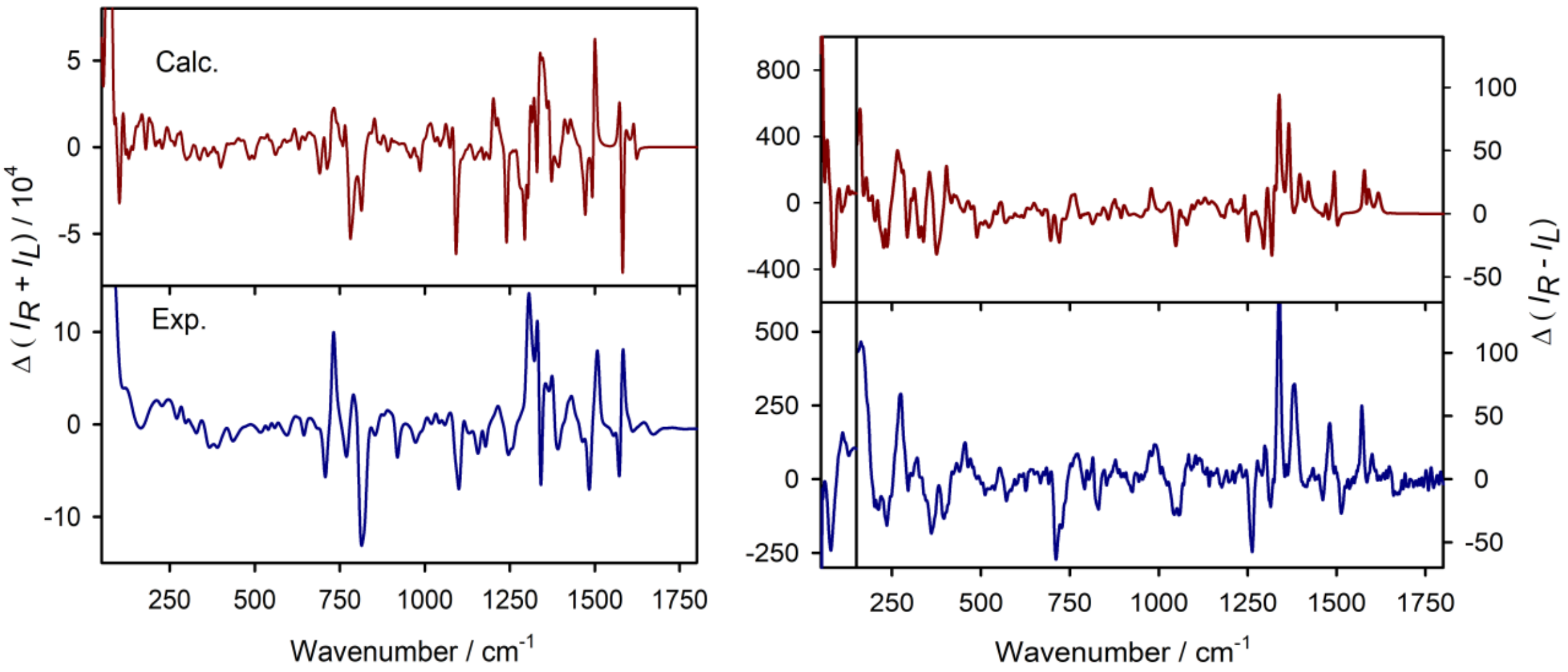

Figure S9. PolyA, differential Raman (left) and ROA (right) spectra, "80°C minus 1°C".

PolyA:

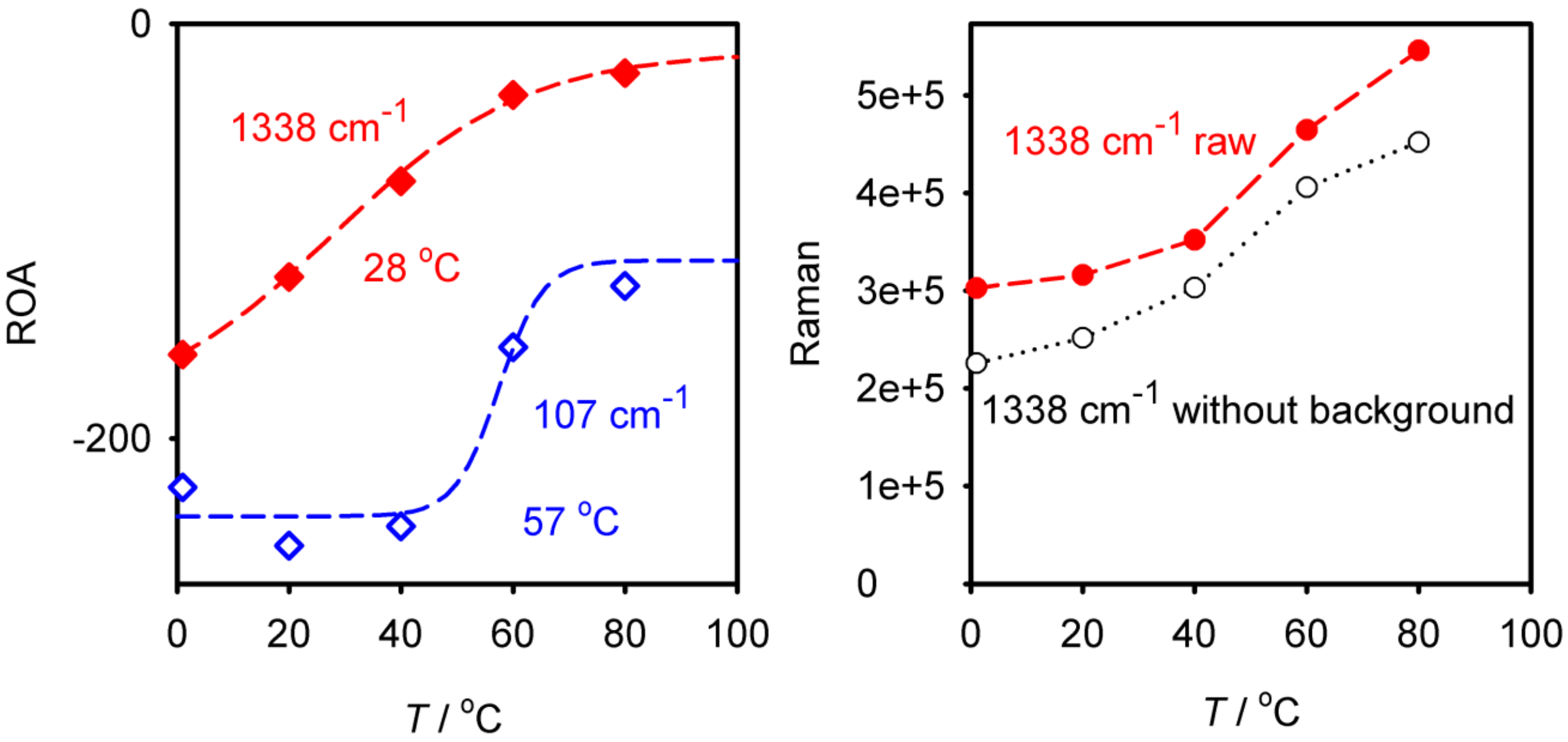

PolyC:

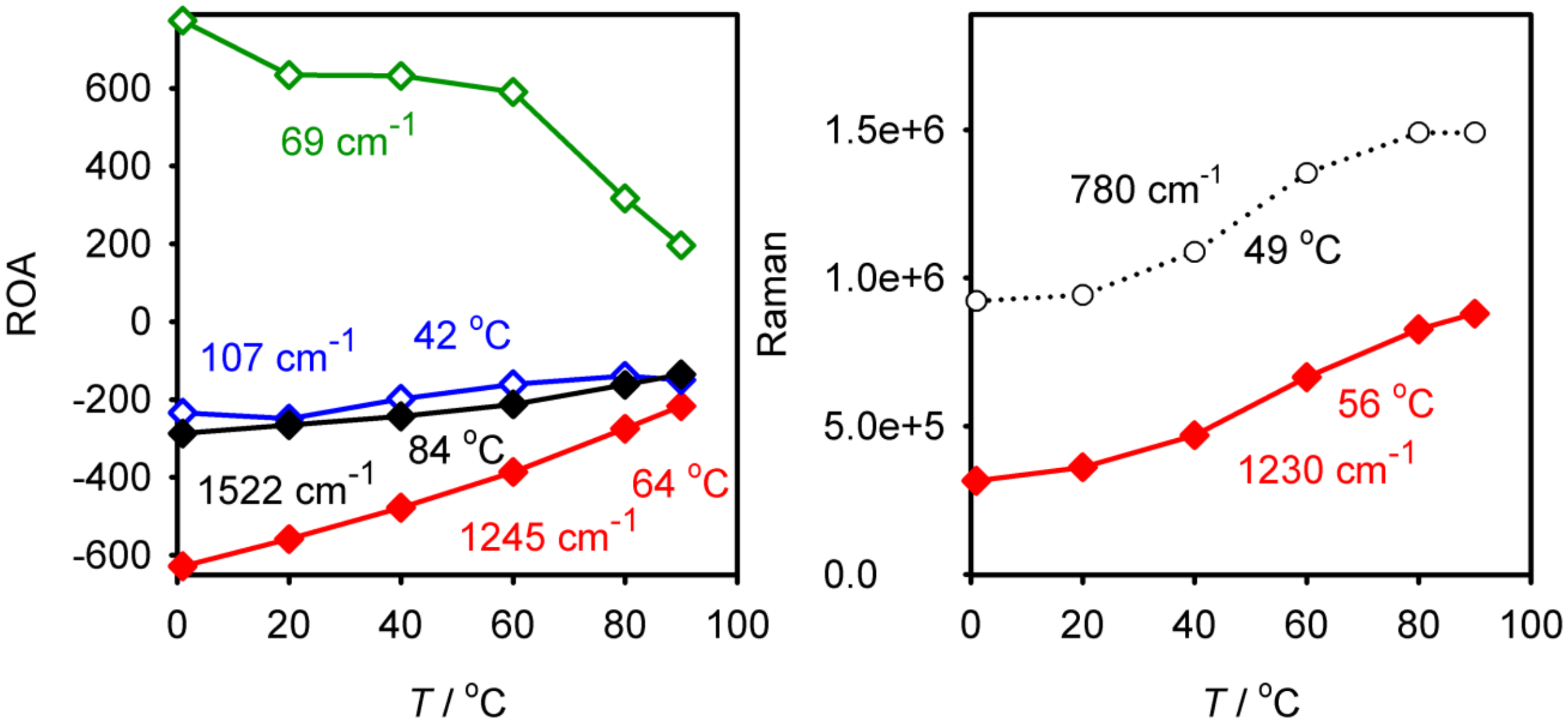

Figure S10. Example of "melting curves", dependence of selected ROA and Raman spectral intensities on temperature, the melting temperatures were obtained from a fit, $S = S_{max} + \frac{S_{min} - S_{max}}{1 + e^{(T-T_m)/d}}$.

.

.

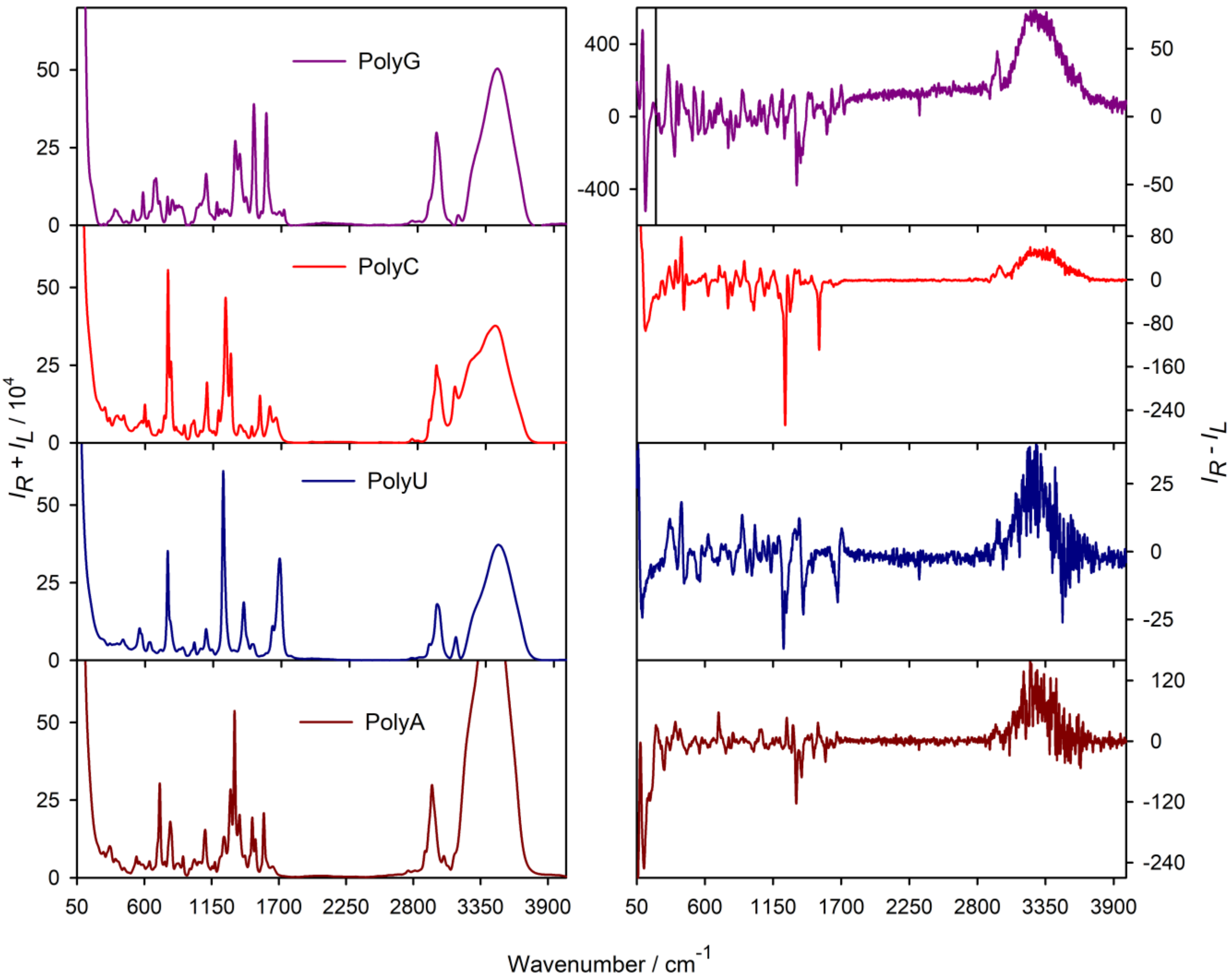

Figure S11. Experimental Raman and ROA spectra of the four polynucleotides, at 20 °C, full wavenumber range.

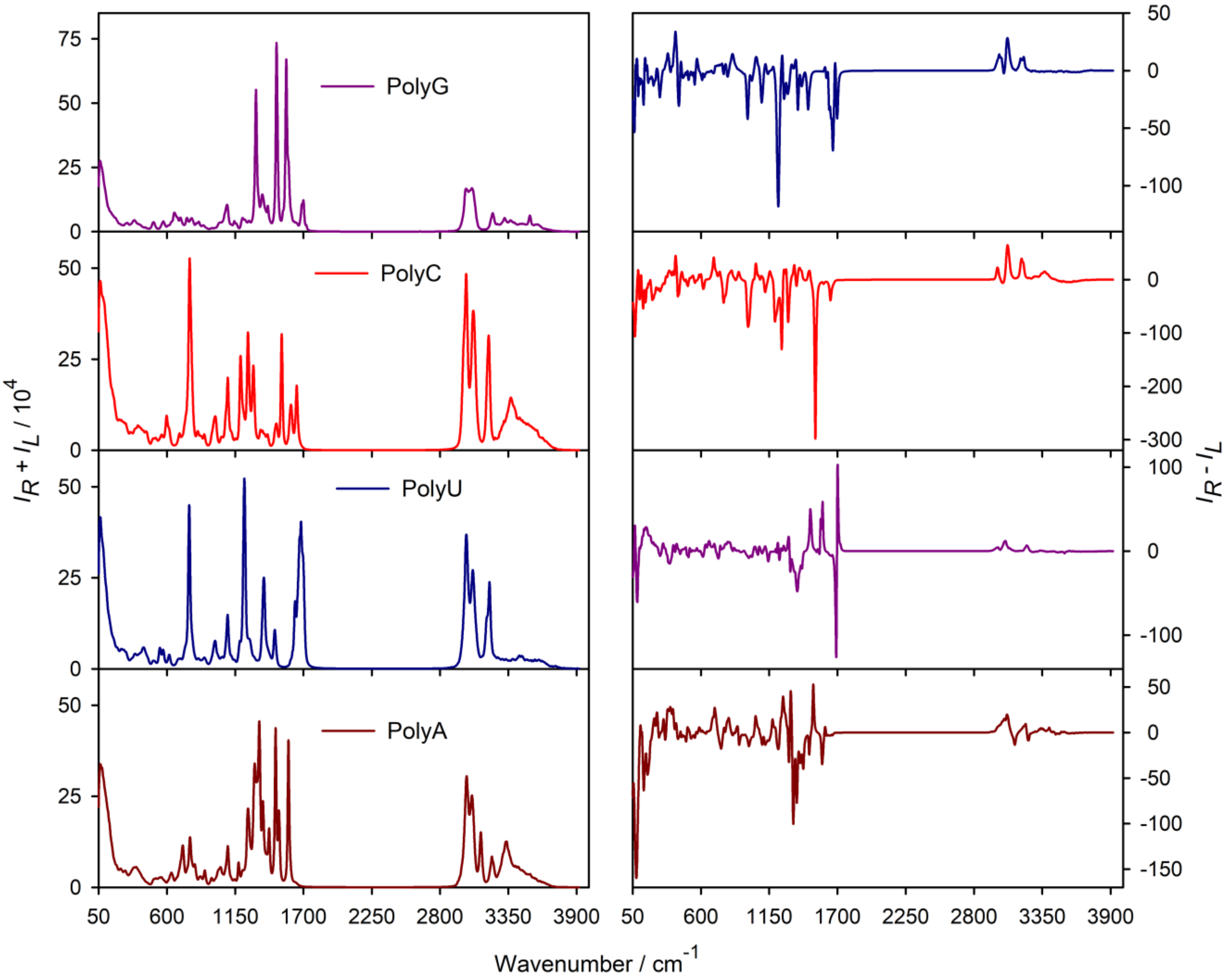

Figure S12. Calculated Raman and ROA spectra of the polynucleotides, full wavenumber range. Each spectrum is an average of 100 MD snapshots, for explicit solvation and 20 °C.

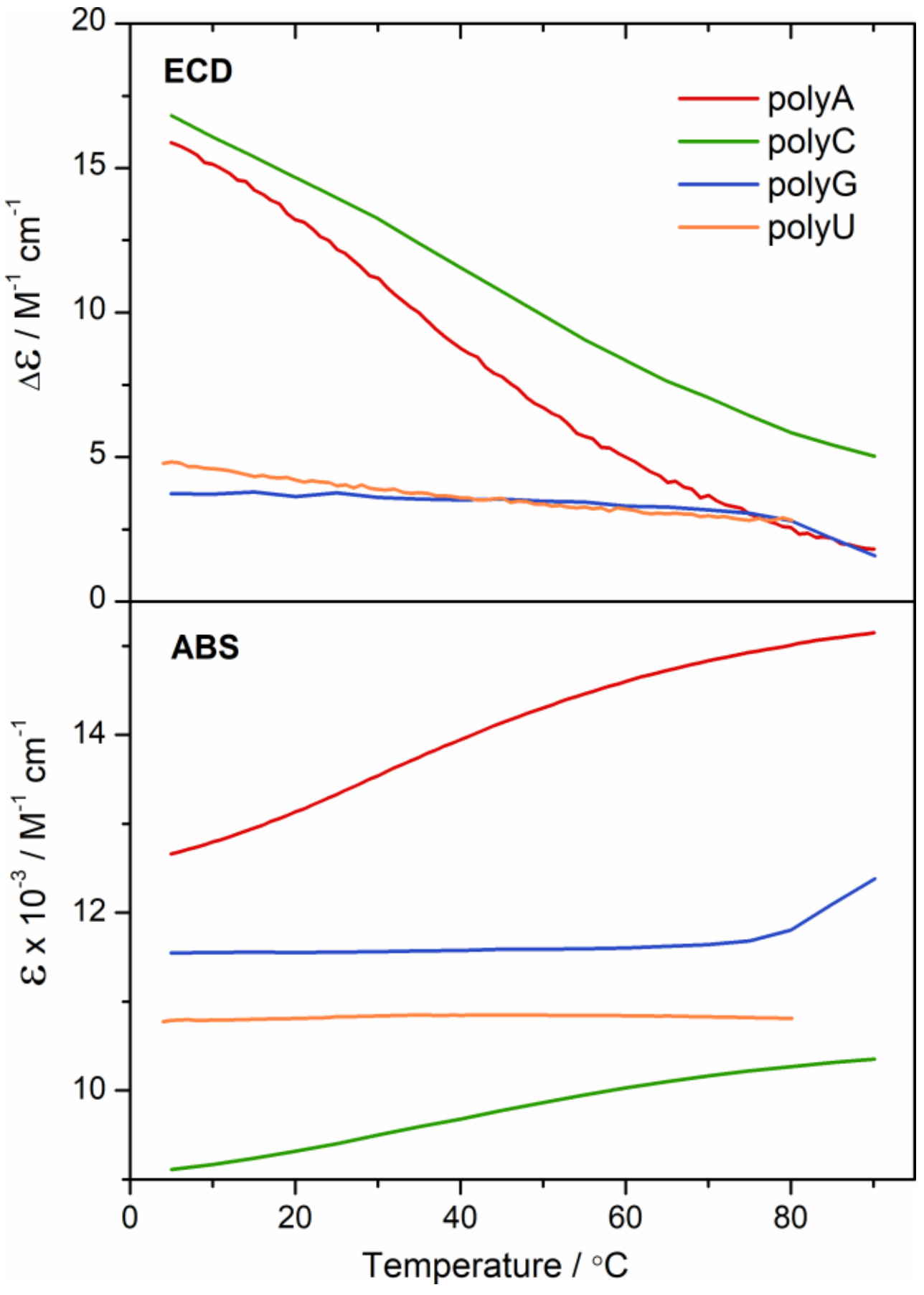

Figure S13. "Melting curves" obtained from UV absorption (bottom) and ECD (top) spectra of four studied polynucleotides.

Table S1. Experimental and calculated frequencies (in cm^{-1}) of selected Raman and ROA bands, for spectra at 20°C.

Calculation		Experiment		
Raman	ROA	Raman	ROA	Assignment
**PolyA**				
1636		1649	1666	$\delta(NH_2)$ (scissoring) δ(OH)
1580	1583	1571	1573	ν(C=C), ν(C=N), δ(NH) (aromatic ν(C=N))
1503	1506	1508	1514	ν(C=C), ν(C=N), δ(CH), δ(NH)
1477	1476	1480	1480	base δ(CH), δ(NH)
1372	1374	1377	1380	ν(C=C), ν(C=N), δ(CH), δ(OH)
1340	1343	1338	1339	δ(CH), δ(OH)
1304	1320	1304	1312	aliphatic δ(CH)
1253	1265	1252	1260	rocking $\delta(NH_2)$, δ(CH), ν(C=N),
1089	1050	1097	1055	symmetric ν(P=O)
904	984	916	992	in plane base deformation
787	819	810	827	
722	760, 703	726	766,709	base breathing
634		641		out of plane base deformation
533	551	536	557	in plane base deformation
	427		455	
	390		399	
	348		361	
	260		270	
	201		208	
	84		108	
**polyU**				
1695	1693	1686	1704	symmetric ν(C=O)
1669				ν(C=O), δ(NH), ν(C=C), ν(C=N)
1632	1667	1629	1672	ν(C=C), δ(CH), , δ(NH)
				ν(C=C), ν(C=N), δ(CH), δ(NH)
1471	1466	1471	1468	δ(CH), δ(NH), base deformation (or ν(C=C), ν(C=N))
1379	1382	1396	1395	δ(CH), δ(NH), base deformation
1234	1224	1231	1235	base δ(CH), δ(NH),
1097	1089	1093	1133	symmetric ν(P=O)
988	985	997	979	in plane base deformation
78-	782	783	826	base, sugar
620		637	626	base, sugar deformations
544-565	536	536	548	in plane base deformation
407	440	421	434	δ(C=O), base out of plane deformation
	409		387	
	315		317	
	95		-	
	58		-	

**polyC**				
1646	1643	1656	1639	ν(C=O), δ(NH_2), ν(C=C)
1606		1607		ν(C=O), δ(NH_2)
1525	1523	1527	1522	ν(C=C), δ(CH), δ(NH)
1481		1461		δ(CH), δ(NH), ν(C=N)
1368	1346	1367	1340	δ(CH), δ(NH)
1296	1303	1292	1287	δ(CH), δ(NH), ν(C=N)
1251	1249	1250	1250	δ(CH), δ(NH),
1195	1155	1193	1154	antisymmetric ν(P=O), base breathing, δ(CH), δ(NH),
1090	1082	1099	1081	sugar base deformation
988	1044	993	1048	symmetric ν(P=O), sugar-phosphate motions
	981		994	
900	904	916	921	sugar, base
	847		852	
	808	807	820	
783	786	785	787	base breathing
	706		716	
	619		627	
594	595	598	600	in-plane base deformation
	413		431	
	392		409	
	341		364	
	309		312	
	252		277	
	140		121	
**polyG**				
		1721	1722	ν(C=O), δ(NH)
1696	1705		1698	
1561	1578	1578	1622	ν(C=C), ν(C=N), δ(NH)
1485	1485	1478	1482	ν(C=C), δ(C=N), δ(NH), aromatic δ(CH)
1412	1374	1414	1448	δ(CH), δ(NH), ν(C=N), ν(C=C)
1366		1364	1370	δ(CH), δ(NH), ν(C=N), ν(C=C)
	1321		1339	
1316	1311	1329	1282	δ(CH), δ(NH)
	1233, 1218		1240, 1221	
1142	1122	1180	1135	
1083	1087	1092	1100	
	1032		1037	
853	872	820	820	
	-		826	
	-	781	784	
707	720	715	724	
		685		
571	575	583	579	
493	499	504	512	
	-		405	
	-		370	
334	336	357	351	
	290		301	
	-		180	
			118	
			94	

Table S2. P-O, C-O, and P=O stretching frequencies of dimethylphosphate[-] anion calculated at several approximation levels.

Method	Basis set	Solvent	ν(P-O), s	ν(P-O), a	ν(P=O), s	ν(C-O), s	ν(P=O), a
HF	6-311++G**	Vacuum	781	799	1178	1233	1398
HF	aug-cc-pVTZ	Vacuum	790	813	1196	1237	1407
HF	6-311++G**	CPCM	786	820	1150	1197	1320
HF	aug-cc-pVTZ	CPCM	797	836	1169	1205	1334
MP2	6-311++G**	CPCM	708	738	1066	1098	1240
MP2	aug-cc-pVTZ	CPCM	722	760	1071	1091	1236
MP4	6-311++G**	CPCM	696	731	1046	1075	1220
B3LYP	6-311++G**	CPCM	684	717	1039	1065	1216
B3LYP [b]	6-31++G**	CPCM/clusters	745	803	1072	1015	1172
B3LYP	aug-cc-pVTZ	CPCM	698	735	1045	1077	1226
B3LYP, anharm.	6-311++G**	CPCM	673	700	1005	1049	1201
exp [a]			754	816	1062	1100	1225
exp [b]			762	829	1087	1038	1205

[a] J. Phys. Chem., 1996, 100 (5), 1559-1568.

[b] J. Phys. Chem. B 2015, 119, 10682−10692.

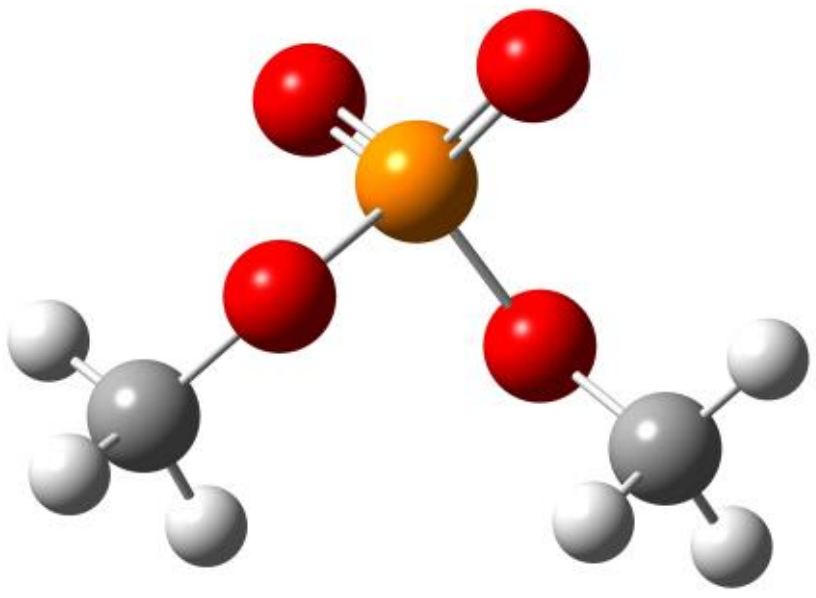

References

1. P. Michal, R. Čelechovský, M. Dudka, J. Kapitán, M. Vůjtek, M. Berešová, J. Šebestík, K. Thangavel and P. Bouř, *J. Phys. Chem. B*, 2019, **123**, 2147-2156.
2. C. J. Wienken, P. Baaske, S. Duhr and D. Braun, *Nucleic Acids Res.*, 2011, **39**, e52-e52.
3. C. Souleil and J. Panijel, *Biochemistry*, 1968, **7**, 7-13.
4. A. G. Petrovic and P. L. Polavarapu, *J Phys Chem B*, 2008, **112**, 2245-2254.
5. A. Sengar, B. Heddi and A. T. Phan, *Biochemistry*, 2014, **53**, 7718-7723.
6. L. Martinez, R. Andrade, E. G. Birgin and J. M. Martinez, *J Comput Chem*, 2009, **30**, 2157-2164.
7. J. Liu, D. Li and X. Liu, *J Chem Phys*, 2016, **145**, 024103.
8. H. M. A. D.A. Case, K. Belfon, I.Y. Ben-Shalom, J.T. Berryman, S.R. Brozell, D.S. Cerutti, T.E. Cheatham, III, G.A. Cisneros, V.W.D. Cruzeiro, T.A. Darden, R.E. Duke, G. Giambasu, M.K. Gilson, H. Gohlke, A.W. Goetz, R. Harris, S. Izadi, S.A. Izmailov, K. Kasavajhala, M.C. Kaymak, E. King, A. Kovalenko, T. Kurtzman, T.S. Lee, S. LeGrand, P. Li, C. Lin, J. Liu, T. Luchko, R. Luo, M. Machado, V. Man, M. Manathunga, K.M. Merz, Y. Miao, O. Mikhailovskii, G. Monard, H. Nguyen, K.A. O'Hearn, A. Onufriev, F. Pan, S. Pantano, R. Qi, A. Rahnamoun, D.R. Roe, A. Roitberg, C. Sagui, S. Schott-Verdugo, A. Shajan, J. Shen, C.L. Simmerling, N.R. Skrynnikov, J. Smith, J. Swails, R.C. Walker, J. Wang, J. Wang, H. Wei, R.M. Wolf, X. Wu, Y. Xiong, Y. Xue, D.M. York, S. Zhao, and P.A. Kollman 2022.
9. J. Kessler, J. Kapitán and P. Bouř, *J. Phys. Chem. Lett.*, 2015, **6**, 3314-3319.
10. M. J. Frisch, G. W. Trucks, H. B. Schlegel, G. E. Scuseria, M. A. Robb, J. R. Cheeseman, G. Scalmani, V. Barone, G. A. Petersson, H. Nakatsuji, X. Li, M. Caricato, A. V. Marenich, J. Bloino, B. G. Janesko, R. Gomperts, B. Mennucci, H. P. Hratchian, J. V. Ortiz, A. F. Izmaylov, J. L. Sonnenberg, D. Williams-Young, F. Ding, F. Lipparini, F. Egidi, J. Goings, B. Peng, A. Petrone, T. Henderson, D. Ranasinghe, V. G. Zakrzewski, J. Gao, N. Rega, G. Zheng, W. Liang, M. Hada, M. Ehara, K. Toyota, R. Fukuda, J. Hasegawa, M. Ishida, T. Nakajima, Y. Honda, O. Kitao, H. Nakai, T. Vreven, K. Throssell, J. A. Montgomery Jr., J. E. Peralta, F. Ogliaro, M. J. Bearpark, J. J. Heyd, E. N. Brothers, K. N. Kudin, V. N. Staroverov, T. A. Keith, R. Kobayashi, J. Normand, K. Raghavachari, A. P. Rendell, J. C. Burant, S. S. Iyengar, J. Tomasi, M. Cossi, J. M. Millam, M. Klene, C. Adamo, R. Cammi, J. W. Ochterski, R. L. Martin, K. Morokuma, O. Farkas, J. B. Foresman and D. J. Fox, *Gaussian 16 Rev. A.03*, (2016) Gaussian, Inc., Wallingford, CT.
11. P. Bouř and T. A. Keiderling, *J. Chem. Phys.*, 2002, **117**, 4126-4132.
12. J. Hudecová, K. H. Hopmann and P. Bouř, *J. Phys. Chem. B*, 2012, **116**, 336-342.
13. A. D. Becke, *J. Chem. Phys.*, 1993, **98**, 5648-5652.
14. S. Grimme, S. Ehrlich and L. Goerigk, *J. Comput. Chem.*, 2011, **32**, 1456-1465.
15. J. Tomasi, B. Mennucci and R. Cammi, *Chem. Rev.*, 2005, **105**, 2999-3093.
16. L. Nafie, *Vibrational optical activity: Principles and applications*, Wiley, Chichester, 2011.
17. P. Bouř, J. Sopková, L. Bednárová, P. Maloň and T. A. Keiderling, *J. Comput. Chem.*, 1997, **18**, 646-659.
18. S. Yamamoto, X. J. Li, K. Ruud and P. Bour, *J. Chem. Theory Comput.*, 2012, **8**, 977-985.
19. V. Andrushchenko and P. Bouř, *Chirality*, 2010, **22**, E96-E114.
20. A. Kurochka, Průša, J., J. Kessler, J. Kapitán and P. Bouř, *Phys. Chem. Chem. Phys.*, 2021, 16635-16645.
